# Patient engagement strategies for adults with chronic conditions: an evidence map

**DOI:** 10.1186/s13643-021-01873-5

**Published:** 2022-03-05

**Authors:** Hanan Aboumatar, Samantha Pitts, Ritu Sharma, Asar Das, Brandon M. Smith, Jeff Day, Katherine Holzhauer, Sejean Yang, Eric B. Bass, Wendy L. Bennett

**Affiliations:** 1 Department of Medicine, Division of General Internal Medicine, Johns Hopkins University School of Medicine, 750 East Pratt Street 15th Floor, Baltimore, MD 21202 USA; 2 Department of Health Policy and Management, Johns Hopkins Bloomberg School of Public Health, Baltimore, MD USA; 3 Department of Pediatrics, Johns Hopkins University School of Medicine, Baltimore, MD USA; 4 Department of Art as Applied to Medicine, Johns Hopkins University School of Medicine, Baltimore, MD USA

**Keywords:** Engagement, Self-management, Shared decision-making, Patient engagement, Family engagement, Self-management support, Evidence map, Chronic disease

## Abstract

**Background:**

Patient and family engagement (PFE) has been defined as a partnership between patients, families, and health care providers to achieve positive health care outcomes. There is evidence that PFE is critical to improving outcomes. We sought to systematically identify and map the evidence on PFE strategies for adults with chronic conditions and identify areas needing more research.

**Methods:**

We searched PubMed, CINAHL, EMBASE, and Cochrane, January 2015 to September 2021 for systematic reviews on strategies for engaging patients with chronic conditions and their caregivers. From each review, we abstracted search dates, number and type of studies, populations, interventions, and outcomes. PFE strategies were categorized into direct patient care, health system, and community-policy level strategies. We found few systematic reviews on strategies at the health system, and none at the community-policy level. In view of this, we also searched for original studies that focused on PFE strategies at those two levels and reviewed the PFE strategies used and study findings.

**Results:**

We found 131 reviews of direct patient care strategies, 5 reviews of health system strategies, and no reviews of community-policy strategies. Four original studies addressed PFE at the health system or community-policy levels. Most direct patient care reviews focused on self-management support (SMS) (*n* = 85) and shared decision-making (SDM) (*n* = 43). Forty-nine reviews reported positive effects, 35 reported potential benefits, 37 reported unclear benefits, and 4 reported no benefits. Health system level strategies mainly involved patients and caregivers serving on advisory councils. PFE strategies with the strongest evidence focused on SMS particularly for patients with diabetes. Many SDM reviews reported potential benefits especially for patients with cancer.

**Discussion:**

Much more evidence exists on the effects of direct patient care strategies on PFE than on the effects of health system or community-policy strategies. Most reviews indicated that direct patient care strategies had positive effects or potential benefits.

A limitation of this evidence map is that due to its focus on reviews, which were plentiful, it did not capture details of individual interventions. Nevertheless, this evidence map should help to focus attention on gaps that require more research in efforts to improve PFE.

**Supplementary Information:**

The online version contains supplementary material available at 10.1186/s13643-021-01873-5.

## Introduction

Health care providers, governmental agencies, patient advocates, and insurers are increasingly focusing on patient and family engagement (PFE) [1–4]. Their reasons vary from considering engagement as a goal and patient right (e.g., patient’s right to take part in all decisions affecting them), to supporting PFE as a means toward improving outcomes and reducing costs [5–7]. Early on, engagement was defined as actions that patients must take to “obtain the greatest benefit from the health care services available to them” [8]. This conceptualization was later expanded to incorporate health care professionals’ and systems’ “actions, interventions, and supports” that promote successful engagement [5, 7–9]. Recently, Carmen et al. further defined PFE as patients, families, and health care providers “working in active partnership at various levels”, including direct care, organizational design and governance, and policy-making to help improve health care outcomes [10]. Carman et al. described a continuum of PFE with activities ranging from basic information sharing (e.g., providers offering information during visits and patients providing feedback on health services) to shared authority and co-leadership of improvement efforts [10]. Increasingly, health care systems are making efforts to engage patients and families to improve patients’ outcomes and experiences [1, 11, 12]. Many hospitals have established patient and family advisory councils [11], and PFE strategies have been incorporated into new primary care models including the Patient Centered Medical Home and the Comprehensive Primary Care Initiative of the Centers for Medicare and Medicaid Services [13, 14].

Studies have demonstrated that engaged patients have better outcomes and lower acute care use [15], and that low levels of engagement are associated with more adverse events [16]. Despite its benefits, not all patients have the capacity to become engaged in their care. Family-caregiver engagement is particularly important to support vulnerable patients including children, the elderly, people at the end of life, and people with disabilities [17, 18].

PFE is particularly relevant for patients with chronic conditions [19]. About 60% of adults in the USA have at least 1 chronic condition, and 4 in 10 adults have two or more [20]. The Chronic Care Model emphasizes the need for an “informed activated patient” to improve outcomes [19, 21]. The increasing complexity of health care places more demands on these patients (e.g., to handle multiple appointments, choose health insurances, make treatment decisions, and self-manage chronic conditions). Supporting patients to meet their goals is consistent with delivering patient-centered care [19]. Engagement strategies that have been studied among people with chronic conditions include self-management support (SMS) interventions, such as programs that enable patients to work with nurses and diabetes educators to advance their glucose monitoring and medication management skills [22–28]; technology-based solutions, such as patient access to the electronic health record and facilitating communication with providers through patient portals [29, 30] and interventions to improve clinicians’ communication skills and shared decision-making (SDM) [31–35].

Health care providers and health systems are particularly invested in identifying and advancing effective PFE strategies to support patients with chronic conditions and improve their health outcomes. However, there are no clear guidelines or summary evidence to inform decision-making by health system leaders on which strategies to deploy to achieve successful engagement and improve outcomes for patients**.** To help address this gap, the Agency for Healthcare Research and Quality (AHRQ) commissioned a review to create a map of the evidence on PFE strategies used to help patients with chronic conditions and identify areas in need of further research.

## Methods

Given the wide range of PFE strategies being researched, we used an evidence mapping approach. Unlike other systematic reviews, evidence maps tackle broad questions and provide a framework to understand key components of the strategies of interest [36–38]. Rather than focusing on describing specific studies, evidence maps use tables and visuals to summarize evidence and enable policy-makers to then refer to specific studies [36–38].

The full-review protocol, methods, and evidence map are described elsewhere [39]. In this paper, we focus on the findings for adults with chronic conditions.

Our review questions were the following:

1. What engagement strategies have been studied to help patients, families, and caregivers manage their chronic conditions and improve patient health outcomes?
2. What gaps exist in the current research?

To inform our approach, we conducted meetings with key informants, including patients, caregivers, providers, insurers, and researchers, and sought their perspectives on the protocol.

### Conceptual framework

We adapted a PFE framework developed by Carman et al. that categorized PFE strategies according to whether they focused on engaging people at the direct patient care , health system, or community-policy levels (Fig. S1) [5]. While a PFE strategy at direct patient care level helps engage patients in their own care, a health system level strategy engages patients in efforts that have an impact beyond their own health care, such as improving health care quality, and a community-policy level strategy engages patients in developing health care policies [5]. According to this framework, robust engagement at all levels is necessary to improve patient outcomes. Engagement at the health system and community-policy levels contributes to development of more patient-centered health care delivery. In turn, patients, caregivers, and providers are able to work effectively together to address system-level barriers that undermine patient’s ability to follow treatment plans and adopt the recommended health behaviors to improve outcomes. Table 1 provides the definitions we used in this review.

**Table 1:** Definitions

Patient and Family Engagement (PFE)	Patients, families, their representatives, and health professionals working in active partnership at various levels across the health care system—direct care, organizational design and governance, and policymaking—to improve health and health care” [ 5 ]. In addition, we used the term PFE to represent engagement of the patient and family, as well as non-family caregivers, who the patient deems part of his or her care.
Chronic diseases	Conditions that last 1 year or more and require ongoing medical attention or limit activities of daily living or both.”
PFE levels	Direct patient-care level strategy is a strategy that directly inform the patients’ own treatment decisions, health behaviors, or outcomes; a health system level strategy is one that engages patients in efforts that have an impact beyond their own health care such as improving health care quality; community-policy level strategy is one that engages patients, consumers, or citizens in policymaking or that engages communities in health care policies.
Benefits categorization for reported findings	‘Positive effects’ describe a study where the authors made clear unequivocal statements about an overall positive effect of the reviewed interventions; ‘potential benefits’ reported when the authors mentioned likely benefits; ‘unclear benefits’ when the authors were inconclusive; and ‘harms’ when the authors reported harm from the reviewed interventions.

We defined the eligibility criteria for studies using the PICOTS (Population, Intervention, Comparison, Outcomes, Timing, and Setting) framework (Table 2) [39].

**Table 2:** Inclusion and exclusion criteria

PICOTS	Include	Exclude
**Population**	• Patients with chronic medical conditions • Patient and family members of committees/councils aimed at improving care • Subpopulations, including • Ethnic and racial minority • Limited language skills • Low literacy/low health literacy • Cognitive impairment	None
**Interventions**	• Direct patient level interventions • Practice, health system, and reimbursement interventions • Models under alternative payment mechanisms • Community-level interventions	• One-time education-only (e.g., providing a handout) • Without 2-way interaction or ability for patient to ask questions (e.g., providing access to web-based educational program)
**Comparators**	Any comparator (pre/post, concurrent)	No comparison group
**Outcomes**	• Intermediate outcomes (e.g., behavior change, cost, provider satisfaction, health system level changes) • Patient outcomes (e.g., mortality, quality of life, utilization)	None
**Timing**	All timing • Right after implementation strategy (within 3 months) • Longer follow-up	
**Setting**	All settings where self-management occurs (e.g., home/community/clinic/assisted living)	Non-USA-based studies

We aimed to include a wide variety of PFE interventions, based on previously reported definitions and the multi-dimensional perspectives of stakeholders on this topic. Study designs with comparator groups were included (e.g., pre/post and concurrent designs). Outcomes of interest included both intermediate outcomes (e.g., behavior change, cost, provider satisfaction, health system level changes) and clinical outcomes (e.g., mortality, quality of life, hospitalizations). We excluded direct patient care interventions that were unidirectional or involved “basic consultation” on the PFE continuum [10]. For example, we excluded interventions involving a one-time education handout, or online informational programs that did not allow patients to ask questions. Also, health system and community-policy level interventions that only included patients and family members as study subjects (e.g., participants in focus groups and surveys) were not considered PFE interventions.

### Data sources and searches

Given the large body of evidence, we focused our search on systematic reviews, with supplemental searches for original research articles in areas having a paucity of reviews. We included search terms for patient/family/consumer engagement, participation, involvement, activation, or empowerment, as well as terms describing engagement interventions based on the study’s conceptual framework and prior publications in this area (Table S1—Search strategy). We searched the following databases; PubMed, CINAHL, EMBASE, and Cochrane from January 2015 through September 2021 for systematic reviews. Because the majority of reviews on PFE strategies focused on direct care level, we conducted another search (using the same terms) to identify relevant original studies on health system and community-policy level strategies. We screened the search output (title/ abstract then full text) and included original studies on PFE strategies at the system and community levels.

### Study selection

Search results were screened independently by two team members, first at the abstract level and then at the full-text level. Discrepancies were resolved by consensus, or by a third reviewer if consensus could not be achieved. To be included, we required systematic reviews to address the research question, be published after 2015, and provide details of their search strategy. For original research articles we excluded those without comparison groups.

### Data extraction and synthesis

The included systematic reviews were reviewed by two team members. Author, publication year, search dates, number and type of included studies, populations, intervention characteristics, quality assessment, measured outcomes, and findings from each eligible review were abstracted. If the two reviewers disagreed, conflicts were resolved by discussion and consensus . PFE strategies were categorized into those at the direct patient care, health system, and community-policy levels, and for each study the reviewers identified whether it reported positive effects, potential benefits, unclear benefits, no benefits, or harms (see Table 1 for definitions). An evidence table was compiled with the characteristics of each included systematic review and its reported findings. Data was then depicted in summary tables that reported on studied chronic conditions, tested interventions, and reported outcomes.

A similar process was followed to abstract information from the included original articles. An evidence map was then constructed to visually represent available evidence. The map along with the summary tables for systematic reviews helped reveal where evidence is most abundant and where it is lacking.

## Results

**Fig. 1:**
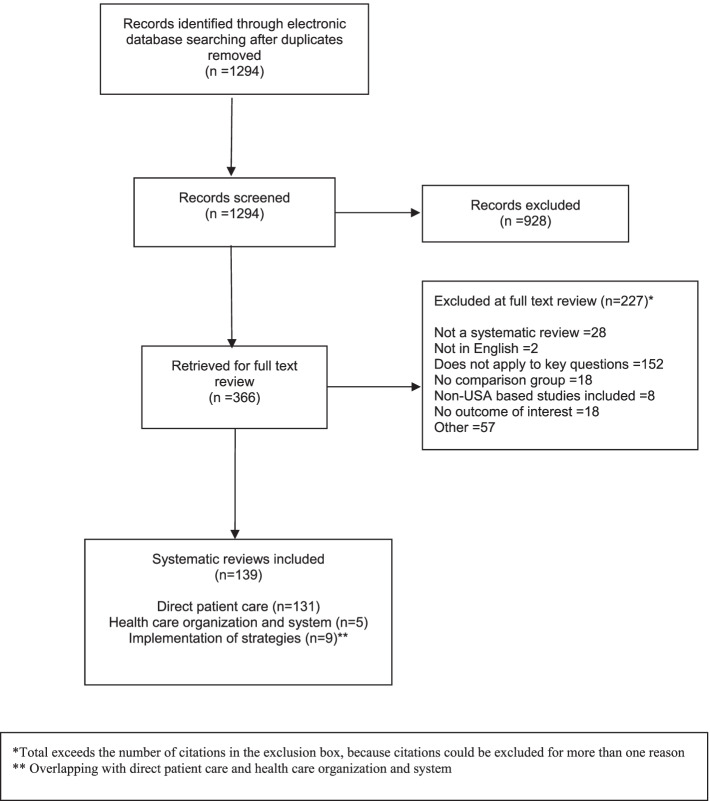
PRISMA flow diagram

The systematic review search identified 1294 references and 366 references were selected for full-text review. Of those 139 systematic reviews reported PFE strategies among adults with chronic conditions (Fig. 1). The original articles search identified 8192 references and 280 references were selected for full-text review. Of those 3 were original articles of PFE strategies at the health system level and one at the community-policy level (Fig. S2).

**Fig. 2:**
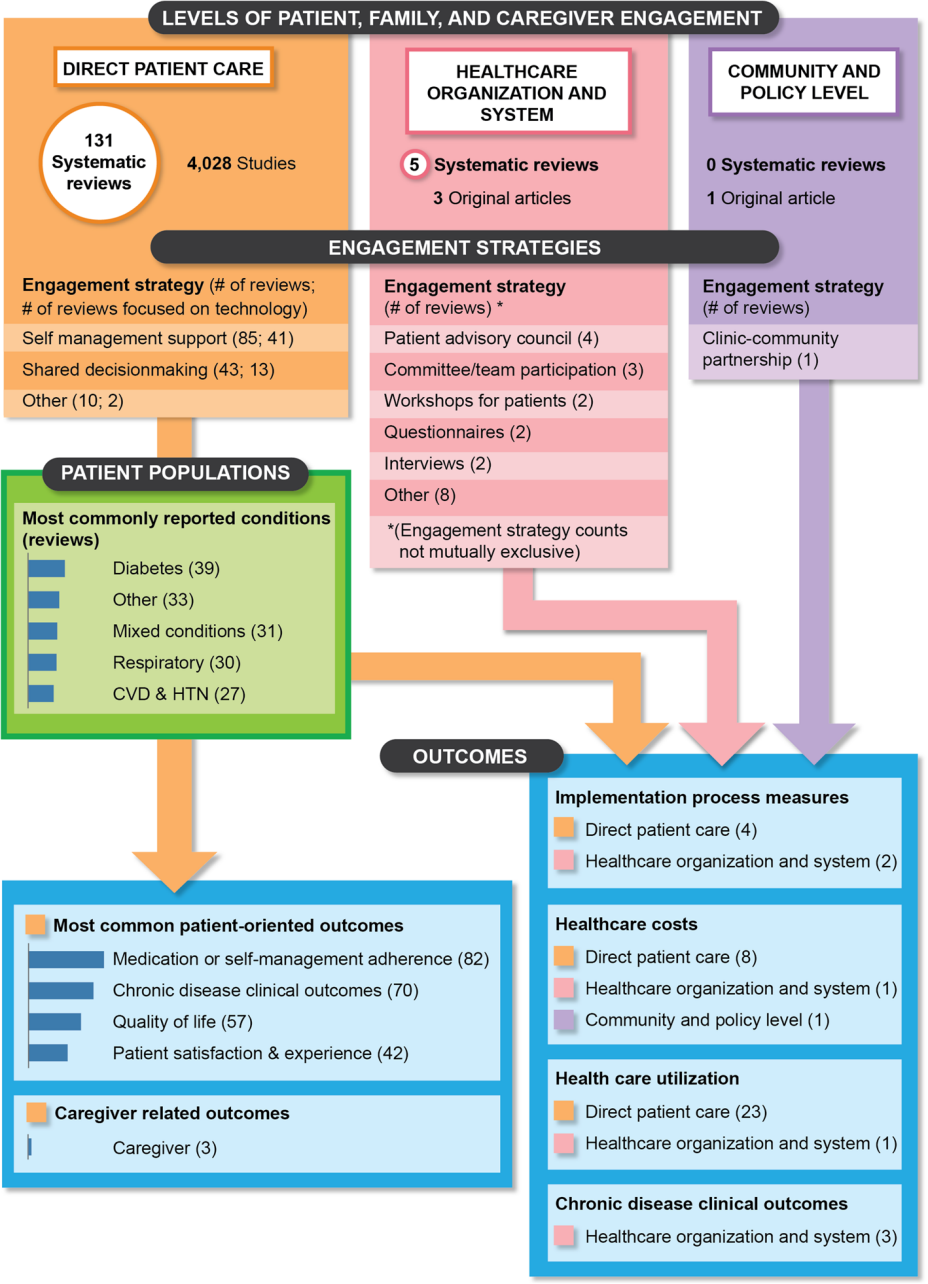
Map of the evidence on patient and family engagement strategies among adults with chronic conditions by level of engagement

Figure 2 maps the available evidence by level of engagement and displays the numbers of systematic reviews of PFE strategies by engagement type (e.g., SMS, SDM), chronic condition, and measured outcomes. Overall, 131 reviews focused on the direct patient care level of engagement, five on the health system level, and none on the community-policy level.

### PFE at the direct patient-care level

Out of the 131 reviews on direct patient care level engagement strategies, 85 focused on SMS, and 43 focused on SDM or enhanced patient-provider communication. Table S2 depicts characteristics of all included systematic reviews at direct care level by intervention type. Table 3 depicts the chronic medical conditions targeted in the direct patient care reviews, by intervention modalities and reported outcomes.

**Table 3:** Chronic medical conditions targeted in systematic reviews, intervention modalities, and reported outcomes by intervention type

Interventions (no. of reviews)*	Clinical focus (no. of reviews)	Outcomes reported (no. of reviews)	Intervention modality (no. of reviews)
Self-management education and support (85)	- Multiple conditions** (9) - Mix of conditions included (22) - Diabetes (34) -CVD and hypertension (23) - Respiratory (27) - Cancer or cancer screening (12) - Mental health (12) - Other (23)	- Chronic disease clinical outcomes (56) - Decisional support outcome (3) - Health care utilization (18) - Medication or self-management adherence (65) - Mortality (2) - Patient satisfaction/experience (20) - Quality of life (38)	- Community health worker/patient navigator (1) - Education/counseling (11) - Mobile health (15) - Nurse/case managers (24) - Patient portal (8) - Peer/lay support (7) - Team-based care (1) - Telehealth (3) - Multiple modalities (13) - Multiple technologies (12) - Other technology (3) - Other (2)
SDM(43)	- Multiple conditions**(5) - Mix of conditions included (11) - Diabetes (4) - CVD and hypertension (2) - Respiratory (2) - Cancer or cancer screening (13) - Mental health (4) - Other (8)	- Chronic disease clinical outcomes (13) - Decisional support outcome (36) - Health care utilization (4) - Medication or self-management adherence (18) - Mortality (0) - Patient satisfaction/experience (22) - Quality of life (15)	- Education/counseling (16) - Mobile health (1) - Nurse/case managers (10) - Patient portal (2) - Telehealth (1) - Multiple modalities (13) - Multiple technologies (2) - Other technology (8) - Other (8)
Other (10)	- Multiple conditions** (1) - Mix of conditions included (2) - Diabetes (2) - CVD and hypertension (2) - Respiratory (2) - Cancer or cancer screening (0) - Mental health (1) - Other (2)	- Chronic disease clinical outcomes (4) - Decisional support outcome (2) - Health care utilization (3) - Medication or self-management adherence (2) - Mortality (2) - Patient satisfaction/experience (2) - Quality of life (6)	- Education/counseling (6) - Nurse/case managers (2) - Peer/lay support (2) - Multiple modalities (5) - Other technology (1) - Other (3)

*Six reviews addressed more than one intervention type; **Multiple conditions refer to reviews of patients who have multimorbidity/comorbidity, *CVD* = cardiovascular disease

Fifty-seven of the 131 reviews focused on studies using mobile health, electronic health record tools, or web-based programs for PFE. Diabetes mellitus was the most studied chronic condition.

Table 4 depicts findings on the benefits of direct PFE, by intervention type and health condition. The reviews that most frequently reported benefits addressed SMS for diabetes and cardiovascular disease, and reviews of SDM for cancer screening and treatment.

**Table 4:**
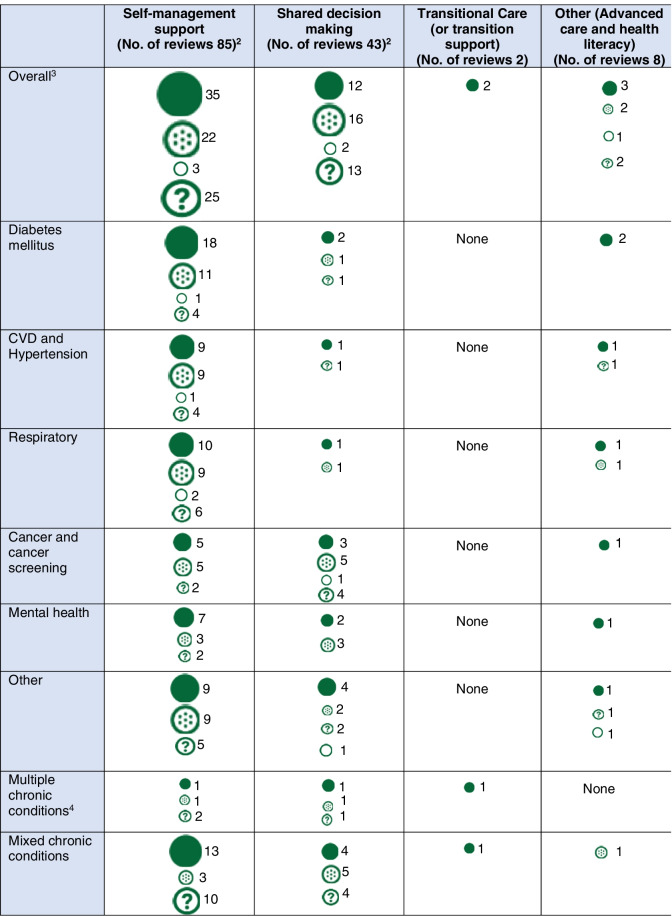
Benefits of patient and family engagement interventions at the direct care level, as reported in systematic reviews, by engagement type and health condition

*Interpretation*:  Positive effects  Potential benefits  No benefits  Unclear benefits

^1^ Circle size is based on total number of reviews

^2^Unclear benefits—in five reviews of self-management and 1 review of shared decision-making, the review question was not about evaluating outcomes (see Table S2 Reviews # 22, 105, 160, 495, 352, 545)

^3^Total exceeds the number for overall because reviews could be included for more than one condition

^4^Multiple conditions refer to reviews of patients who have multimorbidity/comorbidity

#### PFE strategies including Self-management support

We identified 85 reviews evaluating SMS strategies for adults. The number of original studies in the reviews ranged from 2 to 488, with 0 to 105 being randomized controlled trials. The most frequently reviewed chronic conditions were diabetes mellitus (*n* = 34), respiratory disorders (*n* = 27), and cardiovascular disease including hypertension (*n* = 23) (Table 3).

SMS strategies were mostly tested within multi-component interventions. These strategies included (1) education and information sharing on condition and treatment options; (2) helping patients with goal-setting, self-monitoring and symptom management, using action plans, problem-solving, tracking data and feedback; (3) using reminders and alerts, remote monitoring, and decision support to facilitate patient-provider communication and adherence; (4) providing psychosocial support including health care navigation assistance, connection to social services and peers, counseling and cognitive behavioral therapy.

The most frequently reported outcomes were adherence to medication or self-management tasks (reported in 65 reviews), chronic disease control measures (56 reviews), quality of life (38 reviews), patient satisfaction or experience (20 reviews), and health care utilization (18 reviews). Thirty-five reviews reported positive effects, 22 reported potential benefits, 25 reported unclear benefits, and 3 reported no benefits. None reported any harms. Among the 34 reviews focused on SMS in diabetes, 18 reported positive effects and 11 reported potential benefits. These benefits were frequently reported for measures of glycemic control and medication adherence. Cost benefits for SMS interventions were reported in three reviews [40–42].

Two large reviews focused on SMS interventions for low-income, underserved, and minority patients [43, 44]. The first review examined community health center-based interventions for patients with diabetes and reported significant improvement in glucose control when interventions included in-person individual or group education sessions with phone follow-up. SMS interventions that were fully telephone-based showed no significant improvements [43]. The second review focused on community-based health worker interventions among vulnerable populations and reported evidence of cost-effectiveness in self-management of selected health conditions including hypertension and diabetes [44].

Two reviews of studies of patients with multiple chronic conditions reported that SMS had unclear benefits, possibly due to complexity of self-management of multiple chronic conditions and reduced chances for SMS to help these patients [45, 46]. There were three reviews of studies involving caregivers. One of those, included nine RCTs of self-care interventions for patients with various chronic conditions and their caregivers [47], and showed a reduction in rehospitalizations. Another reviewed telehealth interventions involving caregivers and reported better psychological health and reduced burden for caregivers [48]. Seven reviews focused on patient self-management via patient portals and secure messaging systems [49–55]. One of these reviews explored impact on diabetes control (hemoglobin A1c level), and reported that 7 out of 11 included studies demonstrated lower hemoglobin A1c levels with the use of secure messaging [55].

#### PFE strategies including SDM and patient-provider communication

Forty-three reviews evaluated SDM or patient-provider communication. The number of original studies in reviews ranged from 4 to 488 original studies, with 1 to 105 RCTs. The chronic conditions most studied were cancer screening or treatment (*n* = 13), multimorbidity (*n* = 5), and mental health conditions (*n* = 4). SDM interventions often involved multiple components, including provider training and patient education tools, and the interventions used technology-enabled delivery modes and decision supports (Table 2). Frequently reported outcomes were decisional support outcomes (*n* = 36) (e.g., decisional conflict measures), and patient satisfaction or experience (*n* = 22). There were no reports on mortality and few on health care utilization (*n* = 4). Twelve showed positive effects,16 showed potential benefits, 13 showed unclear benefits, and 2 showed no benefits.

A large Cochrane review (*n* = 115 studies) broadly assessed decision aids for people with chronic diseases facing treatment or screening decisions [56]. The review reported high- and moderate-quality evidence of improved knowledge, reduced decisional conflict, more engagement in decision-making, and fewer people choosing discretionary surgery [56].

Four reviews evaluated SDM strategies for advanced care planning and reported a shortage of studies in this area [57–60]. One reported beneficial effects on patients or surrogate decision-makers’ knowledge [58]. Two reviews examined the role of the electronic health record’s patient portal or secure messaging system as a PFE strategy to enhance patient-provider communication [54, 61]. Evidence of benefits were inconclusive. One review reported provider perceptions that releasing abnormal or sensitive test results to patients through the electronic health record could cause confusion or excess worry for patients, but there was no systematic measurement of harm in that review [54]. Cancer screening and treatment were the most studied condition for the SDM reviews, with three reviews showing positive effects [62–64] and 5 reporting potential benefits [65–69]. Two recent reviews reported unclear benefits [70, 71]. One review identified 10 studies focused on cancer treatment decision-making specifically for patients from racial and ethnic minority backgrounds, and showed improved patient engagement outcomes (i.e., higher satisfaction, improved communication) [64] (Table S2).

#### Other direct care PFE strategies

We identified six reviews that focused on engagement around care transitions [72, 73], personalized care planning [74, 75], educational programming for patients undergoing peritoneal dialysis [76], and digital health coaching [77]. One other review examined health literacy and patient activation elements of self-management interventions for patients with COPD [78].

### PFE at the health system level

We identified five reviews [79–83] and three original articles [84–86] that focused on PFE at the health system level. Most commonly addressed chronic conditions were mental health [79, 81, 83], cancer [81, 83], diabetes mellitus [79, 81], and neurologic conditions [81, 83]. The most commonly reported strategies to engage patients at the system level included assembling patient and family advisory councils [79, 81–83], and including patients on committees [81–83], and in forums and workshops [80, 81] and involving patients as educators for medical trainees [80]. Report on impact of PFE at system level were limited to changes in the process of care, policies, documents, and tools. Sharma and colleagues conducted a review of the impact of patient advisors [83]. They reported impact on clinics’ priority setting in one cluster RCT and found no studies with robust designs that reported on clinical outcomes or patient satisfaction [83]. Another review of patient and family advisory councils by Oldfield et al also reported a “paucity of RCTs or high-quality observational studies” [79].

### PFE at the community-policy level

We did not find any reviews on PFE engagement at the community-policy level. We identified one original article that described community-policy level engagement using community advisory councils at the Indian Health Service [87].

### Gaps in evidence

Figure 2 provides an evidence map that highlights the overall findings about the existing evidence. Compared with direct patient care strategies, fewer reviews addressed health system level strategies (*n* = 5) and even when we augmented the search to identify original studies, very few studies (*n* = 3) met our inclusion criterion of having a comparison group. No reviews were found on community-policy level engagement. Five reviews focused on PFE strategies for advanced care planning, and reported inconsistent conclusions [57–60, 75]. Only one review focused on interventions that address health literacy elements, and this review was restricted to SMS interventions for patients with COPD [78]. Few direct patient care reviews focused on patients with multiple chronic conditions (*n* = 15), and vulnerable populations such as urban or rural, minority, low income, or older adults (*n* = 15). Few reviews reported on caregiver outcomes, health care utilization, or cost.

## Discussion

Increasingly, patients and caregivers play key roles not only in managing their own health and health care, but also in contributing to health care system improvement. Given the broad and multi-dimensional scope of PFE, it is not surprising that we found a great deal of heterogeneity among included studies. This is consistent with the recent position paper by the American College of Physicians, “Principles for Patient and Family Partnership in Care,” which highlighted that the meaning of PFE vastly differs by setting, scale, and intended outcomes [1].

This study was conducted to address the information needs of health care leaders on PFE. Given its broad scope, we provided a high-level examination of the evidence on the effects of PFE strategies across a range of chronic diseases and at various levels for engagement. Our findings are consistent with earlier reviews on benefits of PFE strategies at the direct care level on patient outcomes. The evidence map reinforces that there is a need for more high quality evidence on the impact of PFE on health care costs, and the impact of PFE at the health system and policy levels [81, 88, 89].

The evidence map showed that most of the evidence for PFE is at the direct patient care level, with the most studied interventions being on SMS and SDM. The reviews varied in terms of studied chronic diseases (diabetes was the most studied), and clinical settings and modalities for engagement. Strategies with the most frequently reported evidence for effectiveness were SMS interventions, particularly among patients with diabetes. Despite the large number of reviews, we identified inconsistent findings for the benefits of SMS and SDM strategies, sometimes among studies of patients with the same chronic conditions. This is in part due to the heterogeneity of tested interventions, different measures used, and varying quality of the original studies. Most measured anticipated outcomes of engagement including adherence to chronic disease self-management behaviors, clinical outcomes, health care utilization, and patient satisfaction and experience. Measurement of the engagement process and the extent of patient engagement in studied interventions were largely lacking. The latter requires use of validated PFE measures that are specific for patients with various chronic conditions. The need for development of more measures in these areas is increasingly being recognized [89–92]. Although the PFE interventions in principle aim to advance engagement, the lack of assessment of the engagement process makes it impossible to fully assess the merits of various interventions. Furthermore, the extent of engagement likely affects achievement of intended outcomes from studied interventions and may explain the inconsistency in reported findings among studies [9, 93].

The evidence map identified 46 reviews focused on using technology as part of the engagement strategy. The use of patient portals was influenced by patients’ age, ethnicity, education level, health literacy, health status, provider endorsement, and portal usability [61]. More studies are needed that explore ways by which technology can be leveraged in a manner that addresses barriers to its use by patients.

Few studies reported on caregiver measures, which reflected the overall paucity of studies looking at effects of PFE on caregivers. We also found few reviews on decision-making for older adults, advanced care planning and end of life care, compared to other areas of PFE suggesting the need for more focus on this area. Reports on cost-effectiveness of PFE strategies were sparse. More evidence in this area is essential for health care leaders and policy-makers to make the financial case for funding of programs that advance PFE within health systems.

To our knowledge, this is the first systematic review to address PFE strategies focused on health system and community-policy level strategies, in addition to direct patient care level strategies. Similar to our review, the systematic review by Coulter and colleagues included direct-patient care engagement strategies and identified many studies focused on SMS, as well as clinical decision-making through patient-provider communication strategies [9]. Sharma and colleagues conducted a review of PFE strategies specifically related to patient safety and identified only one review with a health system level engagement strategy [93]. One of the principles of the learning health system is to “promote the inclusion of patients as vital members of the learning team,” making it important to identify best practices and high-quality evidence to select strategies that not only engage patients but also lead to improvements in care quality and value [94]. Despite the enthusiasm about increasing patient engagement at a system level and calls to make it an expectation, our evidence map identified a paucity of rigorous studies about the effectiveness and implementation of PFE strategies at the health system level. Though it is not feasible to implement RCTs to assess effectiveness of system level strategies, it is feasible to use other research designs including cluster randomization, stepped-wedge trial, and pre-post designs with comparison groups.

The evidence map highlighted that among various PFE strategies, SMS interventions, particularly for patients with diabetes, have benefits for patients and should be integrated into clinical settings. To accomplish this, health care systems need to promote and provide SMS services for patients with diabetes and other chronic conditions in an equitable manner, addressing social determinants of health and barriers for vulnerable populations (e.g., attention to the needs of patients with low literacy, addressing transportation barriers). Reimbursement policies that enable provision of these services in an equitable and sustainable manner are needed. Furthermore, the evidence map shows that many reviews of SDM interventions, particularly among patients with cancer, showed potential benefits especially for patient satisfaction. More studies are needed that examine interventions that are likely to be successfully implemented and sustained by health care professionals and patients with cancer and other preference sensitive conditions. Studies on SDM with older adults and people living with dementia are also needed.

Our study has several strengths. First, to our knowledge, this is the first review to address PFE strategies at the levels of direct patient care, health system, and community/policy. Second, given the widespread implementation of electronic health records and the proliferation of mobile phone applications in recent years, an important contribution of our map was the identification of many reviews focused on using technology as part of the engagement strategy. Third, this review took a broad and inclusive approach for reviewing PFE strategies in response to the different needs and perspectives of the multiple stakeholders who are interested in PFE and its advancement in clinical settings.

Our study has several limitations. First, given the broad scope of PFE, we focused on synthesizing findings from systematic reviews, rather than from all original studies. For each of the included systematic reviews we reported on the total count of primary studies, the medical conditions studied, the outcomes measured, the intervention types and modalities and the findings. Given the aim and broad scope of this paper, we did not abstract specific information about the tested interventions, which were often not available from systematic reviews. Thus, our study offers an evidence map that allows for health care leaders to quickly identify areas and populations where benefits from PFE interventions have been consistently reported and to consider how they might implement these strategies into their health systems. Second, we excluded articles and reviews explicitly focused on patient engagement in research studies. Third, we may have missed some exemplars of community-policy level engagement, as few of these articles met inclusion criteria for having a comparison group or including outcomes of interest [95–104].

In conclusion, we identified a large body and diversity of evidence on direct patient care level engagement strategies, most of which indicated positive effects or potential benefits. We also found multiple gaps in evidence that call for more research on strategies to engage patients with chronic conditions and their caregivers, especially at the health system and community levels.

## Supplementary Information

**Additional file 1: Figure S1.** Patient, family, and caregiver engagement conceptual framework. **Table S1.** PubMed Search Strategy. **Figure S2**. Search flow diagram for original studies. **Table S2.** Characteristics of included systematic reviews on PFE strategies at the direct patient care level and their findings.

## Data Availability

Not applicable

## Abbreviations

PFE: Patient and family engagement
SMS: Self-management support
SDM: Shared decision-making
RCTs: Randomized controlled trials
AHRQ: Agency for Healthcare Research and Quality
PICOTS: Population, Intervention, Comparison, Outcomes, Timing, and Setting
