# Supplementary material for: Patient engagement strategies for adults with chronic conditions: an evidence map
Source: Syst Rev. 2022 Mar 5;11:39. doi: 10.1186/s13643-021-01873-5 (PMC8898416; doi:10.1186/s13643-021-01873-5)
Supplement: Supplementary file 1 — Additional file 1: Figure S1. Patient, family, and caregiver engagement conceptual framework. Table S1. PubMed Search Strategy. Figure S2. Search flow diagram for original studies. Table S2. Characteristics of included systematic reviews on PFE strategies at the direct patient care level and their findings. [file 13643_2021_1873_MOESM1_ESM.docx]

**Additional file 1**

**Figure S1. Patient, family, and caregiver engagement conceptual framework**


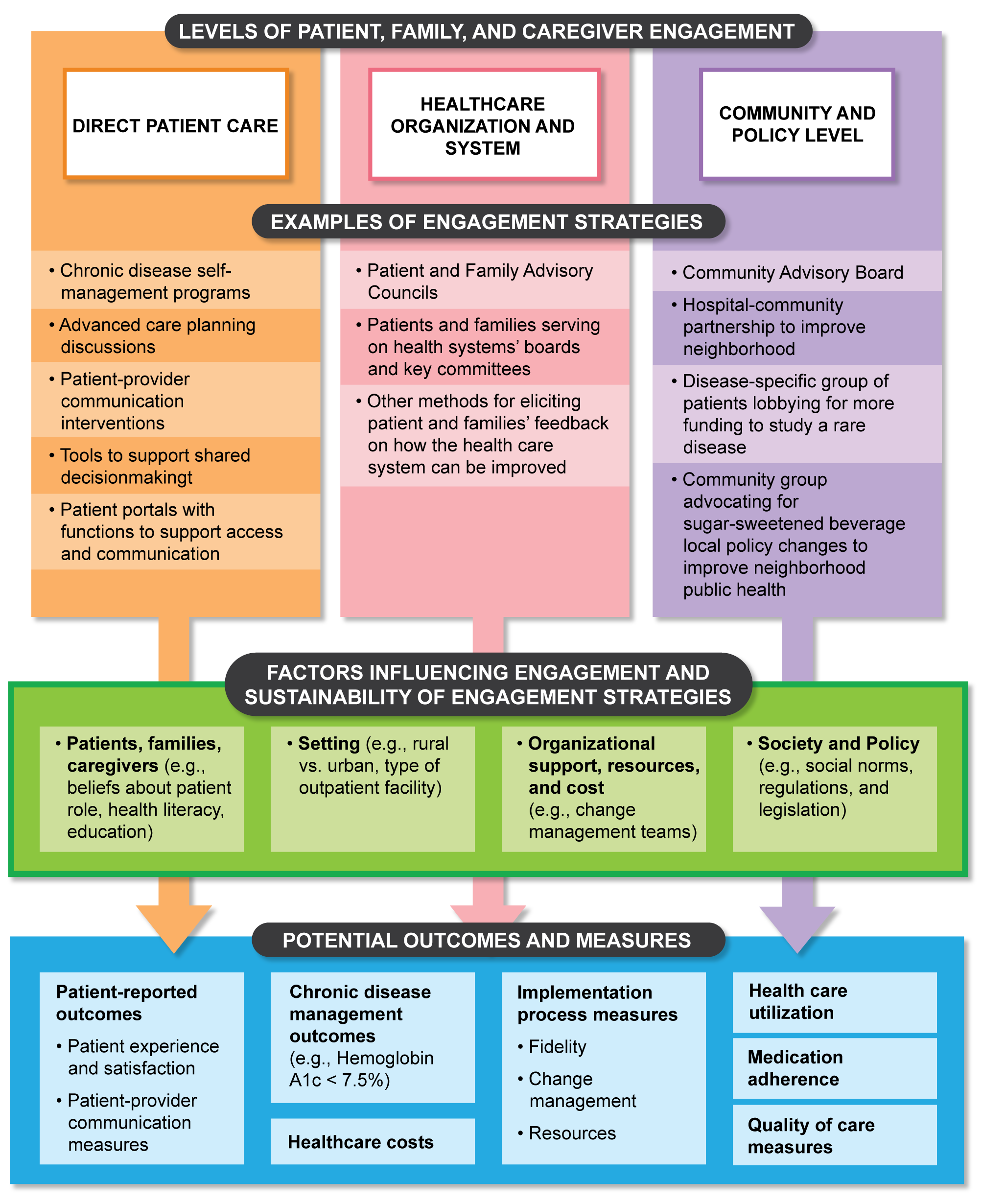


**Table S1. Pubmed Search Strategy**

| **#** | **String** |
| --- | --- |
| 1.  Population | Patient Participation[Mesh] OR “patient participation”[tiab] OR “patient engagement” [tiab] OR “patient involvement” [tiab] OR “patient empowerment” [tiab] OR “patient partnership” [tiab] OR “patient activation” [tiab] OR “patient-activated” [tiab] OR “family participation”[tiab] OR “family engagement” [tiab] OR “family involvement” [tiab] OR “family empowerment” [tiab] OR “family partnership” [tiab] OR “family activation” [tiab] OR “consumer participation”[tiab] OR “consumer engagement” [tiab] OR “consumer involvement” [tiab] OR “consumer empowerment” [tiab] OR “consumer partnership” [tiab] OR “consumer activation” [tiab] OR “caregiver participation”[tiab] OR “caregiver engagement” [tiab] OR “caregiver involvement” [tiab] OR “caregiver empowerment” [tiab] OR “caregiver activation” [tiab] OR “patient context” [tiab] OR “patient capacity” [tiab] OR “patients capacity” [tiab] |
| 2.  Intervention | Advisory Committees [Mesh] OR PFAC [tiab] OR “patient council” [tiab] OR “patient committee”[tiab] OR “patient advisor” [tiab] OR “family council” [tiab] OR “consumer council” [tiab] OR “family advisor” [tiab] OR “advisory council” [tiab] OR “community advisory” [tiab] OR Self-Management [mh] OR “Self-Management” [tiab] OR “Self Management” [tiab] OR Shared Decision Making [mh] OR “decision making”[tiab] OR “decision-making” [tiab] OR “Speaking up“ [tiab] OR telemedicine [mh] OR Electronic Health Records [mh] OR “Electronic Health Records” [tiab] OR "Medical Informatics"[mh] OR "health informatics"[tiab] OR "mobile health" [tiab] OR "eHealth" [tiab] OR "digital health"[tiab] OR "smart phone"[tiab] OR "mobile app"[tiab] OR "mobile applications"[tiab] OR "mHealth"[tiab] OR "smartphones" [tiab] OR Patient Portals [mh] OR “Patient Portals” [tiab] OR Patient Access to Records [mh] OR “Patient Access to Records”[tiab] OR “Opennotes” [tiab] OR Health Literacy [mh] OR literacy [tiab] OR hospice [tiab] OR palliative [tiab] OR “end-of-life” [tiab] OR “end of life”[tiab] OR terminal care [mh] OR Palliative Care [mh] OR “patient safety”[tiab] OR “decision support”[tiab] OR Advance Care Planning [mh] OR “Advance Care Planning” [tiab] OR “Advance Directives” [tiab] OR “peer support”[tiab] OR “social support”[tiab] OR “family support”[tiab] OR “healthcare professional support” [tiab] OR “patient navigator” [tiab] OR Accountable Care Organizations[mh] OR Population Health Management [MH] OR “Accountable Care Organizations”[tiab] OR “Population Health Management” [tiab] |
| 1 AND 2 | ((Patient Participation[Mesh] OR “patient participation”[tiab] OR “patient engagement” [tiab] OR “patient involvement” [tiab] OR “patient empowerment” [tiab] OR “patient partnership” [tiab] OR “patient activation” [tiab] OR “patient-activated” [tiab] OR “family participation”[tiab] OR “family engagement” [tiab] OR “family involvement” [tiab] OR “family empowerment” [tiab] OR “family partnership” [tiab] OR “family activation” [tiab] OR “consumer participation”[tiab] OR “consumer engagement” [tiab] OR “consumer involvement” [tiab] OR “consumer empowerment” [tiab] OR “consumer partnership” [tiab] OR “consumer activation” [tiab] OR “caregiver participation”[tiab] OR “caregiver engagement” [tiab] OR “caregiver involvement” [tiab] OR “caregiver empowerment” [tiab] OR “caregiver activation” [tiab] OR “patient context” [tiab] OR “patient capacity” [tiab] OR “patients capacity” [tiab])) AND (Advisory Committees [Mesh] OR PFAC [tiab] OR “patient council” [tiab] OR “patient committee”[tiab] OR “patient advisor” [tiab] OR “family council” [tiab] OR “consumer council” [tiab] OR “family advisor” [tiab] OR “advisory council” [tiab] OR “community advisory” [tiab] OR Self-Management [mh] OR “Self-Management” [tiab] OR “Self Management” [tiab] OR Shared Decision Making [mh] OR “decision making”[tiab] OR “decision-making” [tiab] OR “Speaking up“ [tiab] OR telemedicine [mh] OR Electronic Health Records [mh] OR “Electronic Health Records” [tiab] OR "Medical Informatics"[mh] OR "health informatics"[tiab] OR "mobile health" [tiab] OR "eHealth" [tiab] OR "digital health"[tiab] OR "smart phone"[tiab] OR "mobile app"[tiab] OR "mobile applications"[tiab] OR "mHealth"[tiab] OR "smartphones" [tiab] OR Patient Portals [mh] OR “Patient Portals” [tiab] OR Patient Access to Records [mh] OR “Patient Access to Records”[tiab] OR “Opennotes” [tiab] OR Health Literacy [mh] OR literacy [tiab] OR hospice [tiab] OR palliative [tiab] OR “end-of-life” [tiab] OR “end of life”[tiab] OR terminal care [mh] OR Palliative Care [mh] OR “patient safety”[tiab] OR “decision support”[tiab] OR Advance Care Planning [mh] OR “Advance Care Planning” [tiab] OR “Advance Directives” [tiab] OR “peer support”[tiab] OR “social support”[tiab] OR “family support”[tiab] OR “healthcare professional support” [tiab] OR “patient navigator” [tiab] OR Accountable Care Organizations[mh] OR Population Health Management [MH] OR “Accountable Care Organizations”[tiab] OR “Population Health Management” [tiab]) |
| 4. Limit  2015 -present | January 2015 -January 2020 |
| 5. Limit to systematic reviews | #4 AND Cochrane validated systematic review search |
| 6. Original articles without systematic reviews | #4 AND without Cochrane validated systematic review search |

**Figure S2. Search flow diagram for original studies**

Records excluded
(n =7912)

Excluded at full text review (n=135)*
Not an original article =7; Not in English =2; Does not apply to key questions =19; No comparison group =16; Non-USA based studies =83; No outcome of interest =12; Other =6

Address only direct patient care (n=141)

Records identified through electronic database searching after duplicates removed
(n =8192)

Retrieved for full text review
(n =280)

Records screened
(n =8192)

Original studies included

(n=4)

Health care organization and system (n=3)

Community and policy level (n=1)

**Table S2. Characteristics of included systematic reviews on PFE strategies at the direct patient care level and their findings**

| **Author, year** | **Review aim** | **Search years** | **Total number of studies** | **Medical conditions** | **Strength of evidence report** | **Systematic review findings category**(positive effects, potential benefits, unclear benefits, no benefits, or harms) | | |
| --- | --- | --- | --- | --- | --- | --- | --- | --- |
| **Self-management Support (Including health behavior change)** | | | | | | | |  |
| Yin,2019[1] | How mobile health apps for IBD care have fit into a clinical care framework and the challenges that clinicians and technologists face in approaching future opportunities. | 2010-2019 | 28 | Mixed conditions, IBD | No | | Positive benefit | |
| Debon,2019[2] | To identify mobile health applications with features for improving the lifestyle of patients with chronic diseases. | Inception-2018 | 24 | Mixed conditions | No | | Positive benefit | |
| Luedke,2019[3] | For adults with epilepsy, i. what are the most commonly employed components of self-management interventions evaluated in comparative studies? Ii. effects of self-management interventions on self-management skills and self-efficacy, clinical outcomes, and health care utilization iii. Barriers and facilitators | Inception-2018 | 28 | Epilepsy | Yes | | Unclear benefit | |
| Warner,2019[4] | To identify whether community-based Self-Management Programs (SMPs) actively engaged, or taught, individuals’ patient-oriented strategies; and whether having these attributes led to significant differences in outcomes. | 1986-2016 | 31 | Mixed conditions, DM, CVD, Respiratory, Osteoarthritis, physical rehab | Yes | | Potential benefit | |
| Han,2019[5] | Effects of community health center interventions in people with diabetes | Inception-2018 | 29 | DM | Yes | | Positive benefit | |
| Levengood,2019[6] | Examine the effectiveness of team-based care in improving health outcomes of people living with diabetes | 2003-2015 | 35 | DM | No | | Positive benefit | |
| Yin,2019[7] | This study aimed to characterize the different tasks and contexts in which context-aware systems for patient work were used as well as to assess any existing evidence about the impact of such systems on health-related process or outcome measures. | Not mentioned -2017 | 6 | DM, CVD, Respiratory, Mental health, Kidney disease, Parkinson disease | No | | Unclear benefit | |
| Lederle,2019[8] | Our review and meta-analysis sheds light on the relationship between lay-led self-management programs and health care utilization. | 2006-2017 | 49 | DM, CVD, Respiratory, Cancer or cancer screening, Mental health, Any chronic disease (lung, heart, arthritis, diabetes, mental illness, stroke, back pain, cancer) | No | | Potential benefit | |
| Nazarov,2019[9] | Identify studies of interventions that support the maintenance of work and return to work (RTW) among workers with chronic illnesses. | Not mentioned -2018 | 15 | DM, CVD, Respiratory, Mental health, Neuro | Yes | | Positive benefit | |
| Sangrar,2019[10] | To review the literature on chronic disease self-management programs that blend face-to-face and online/computer-based education design and delivery. | 2004-2019 | 12 | DM, Respiratory, diabetes, asthma, COPD | No | | Benefit category:  Not applicable | |
| Dounavi,2019[11] | Identify existing evidence on the efficacy of mobile health technology in facilitating weight management behaviors, such as healthy food consumption and physical activity | 2012-2017 | 39 | Obesity | Yes | | Positive benefit | |
| Skrabal Ross,2018[12] | This study aims to explore what is known about mobile phone–delivered interventions designed to enhance adherence to oral chemotherapy, to examine the reported findings on the utility of these interventions in increasing oral chemotherapy adherence | Not mentioned-2018 | 5 | Cancer or cancer screening | No | | Unclear benefit | |
| Lancaster,2018[13] | To determine the impact of patients’ use of eHealth tools on self-reporting adverse effects and symptoms that promote changes to Medication use | 2000-2018 | 14 | DM, Respiratory, Cancer or cancer screening | Yes | | Positive benefit | |
| Lycett,2018[14] | Examine the use and application of theory in the development of digital interventions to enhance asthma self-management and to evaluate the effectiveness of theory-based interventions in improving adherence, self-management, and clinical outcomes | Not mentioned-2017 | 14 | Respiratory | Yes | | Positive benefit | |
| Bashi,2018[15] | To investigate smartphone-based educational interventions and their structures and strategies for patient self-management. | 2006-2016 | 15 | Mixed chronic conditions     Mental health, | Yes | | Positive benefit | |
| Alessa,2018[16] | A systematic review was conducted to assess the effectiveness of apps in lowering blood pressure, as well as their usability and patients’ satisfaction with their use. | 2008-2016 | 21 | CVD | Yes | | Potential benefit | |
| Ko,2018[17] | This review aimed to examine how self-management has been operationalized in the context of multiple chronic conditions. | 2006-2017 | 7 | Multiple chronic conditions | No | | Benefit category:  Not applicable | |
| Changizi,2017[18] | The present study was conducted aiming to assess the effectiveness of mHealth in improving health behaviors among an elderly population | 2012-2016 | 12 | DM, CVD, Obesity | No | | Positive benefit | |
| Kelly,2018[19] | To assess the efficacy, cost-effectiveness and adverse effects of self-management interventions for adults and children with non-cystic fibrosis bronchiectasis. | 1937-2018 | 2 | Respiratory | Yes | | Unclear benefit | |
| Niznik,2018[20] | Identify the impact of clinical pharmacist telemedicine interventions on clinical outcomes, subsequently defined as clinical disease management, patient self-management, and adherence, in outpatient or ambulatory settings | Database inception-2016 | 34 | DM, CVD, Respiratory, Mental health, Kidney disease, hyperlipidemia | No | | Positive benefit | |
| Jeddi,2017[21] | We conducted a systematic review of randomized controlled trials (RCTs) to assess the features and effects of IT-based interventions on self-management outcomes of CKD patients | Not mentioned-2016 | 8 | Kidney disease | Yes | | Positive benefit | |
| Conway,2017[22] | The objective of this integrative review was to examine the types of digital health technologies that targeted Medication adherence in the adult population with diabetes or hypertension | 2006-2016 | 13 | DM, CVD | No | | Unclear benefit | |
| Kim,2017[23] | Scoping review to (1) understand the nature, extent, and range of smart device-based research activities, (2) identify the limitations of the current research and knowledge gap, (3) recommend future research directions | 2010-2016 | 51 | Mixed conditions, DM, CVD, Respiratory | No | | Unclear benefit | |
| Cho,2017[24] | Aim of this SR was to assess the impact of technology-mediated interventions on QoL and to identify the instruments used to measure the QoL of persons living with HIV/AIDS (PLWH). | 1994-2016 | 10 | Persons living with HIV/AIDS (PLWH) | Yes | | Unclear benefit | |
| Clarkesmith,2017[25] | Synthesizing the evidence about the effects of educational and behavioral interventions in patients with atrial fibrillation who are taking oral anticoagulant Medication. | 1806-2016 | 11 | CVD, Patients with atrial fibrillation | Yes | | Unclear benefit | |
| Massimi,2017[26] | The aim of this systematic review and meta-analysis is to assess the efficacy of the nurse-led self-management support versus usual care evaluating patient outcomes in chronic care community programs. | 1990-2016 | 29 | Multiple chronic conditions, DM, CVD | Yes | | Positive benefit | |
| Zomahoun,2016[27] | This SR aimed to assess whether Motivational Interview interventions are effective to enhance Medication adherence in adults with chronic diseases and to explore the effect of individual MI intervention characteristics. | 2012-2016 | 19 | Mixed conditions | Yes | | Positive benefit | |
| Whiteman,2016[28] | Aimed to review the evidence of the effect of self-management interventions targeting both medical and psychiatric illnesses and evaluate the potential for implementation. | 1946-2015 | 15 | Multiple chronic conditions, Mental health, | Yes | | Potential benefit | |
| Zhao,2016[29] | To synthesize the effects of theory-based self-management educational interventions on patients with type 2 diabetes (T2DM) in randomized controlled trials. | 1980-2015 | 20 | DM | Yes | | Positive benefit | |
| Teljeur,2016[30] | To systematically review the evidence on the costs and cost-effectiveness of self-management support interventions for people with diabetes. | Not mentioned-2015 | 37 | DM | Yes | | Positive benefit | |
| Hecke,2016[31] | This SR aims to assess the quality of evidence and determine the effect of patient-related and economic outcomes of selfmanagement support interventions in chronically ill patients with a low socio-economic status. | 2000-2013 | 27 | Mixed conditions, DM | Yes | | Unclear benefit | |
| Hooft,2016[32] | The aim of this study was to examine how nurse-led interventions that support self-management of outpatients with chronic conditions work and in what contexts they work successfully | 2000-2015 | 38 | Mixed conditions | Yes | | Potential benefit | |
| Stenberg,2016[33] | Scoping Review: To give a comprehensive overview of benefits and challenges from participating in group based patient education programs that are carried out by health care professionals and lay participants, aimed at promoting self-management for people living with chronic illness. | 2008-2015 | 47 | Mixed conditions, DM, CVD, Cancer or cancer screening, Mental health | Yes | | Positive benefit | |
| Plow,2016[34] | The purpose of this scoping review was to describe randomized controlled trials (RCTs) of tailored self-management interventions in adults with neurological and musculoskeletal conditions that characteristically result in mobility impairments. we focused on summarizing the outcomes of these RCTs and the strategies used to promote behavior change. | 1980-2015 | 13 | Neuro | No | | Potential benefit | |
| Kew,2016[35] | To assess the efficacy and safety of home telemonitoring with healthcare professional feedback between clinic visits, compared with usual care for asthma | 1992-2016 | 18 | Respiratory | Yes | | Unclear benefit | |
| Sakakibara,2016[36] | To describe the self-management interventions used to improve risk factor control in stroke patients and quantitatively assess their effects overall risk factor control from lifestyle behavior and individual risk factors. | Inception-2015 | 14 | Neurology: Stroke patients only | Yes | | Positive benefit | |
| Bolscher-Niehuis,2016[37] | To gain insight into the evidence of the effects of self-management support programmes on the activities of daily living of older adults living at home. | 1998-2015 | 12 | Mixed conditions | Yes | | Positive benefit | |
| Sokol,2016[38] | To assess the reach and effectiveness of peer support among those who are hardly reached, along with peer support strategies (conceptual and operational) used. | 2000-2015 | 47 | Mixed conditions, Maternal and health issues | No | | No benefit assessment | |
| Whitehead,2016[39] | To assess the effectiveness of mobile phone and tablet apps in self-management of key symptoms of long-term conditions. (Only one RCT from US) | 2005-2016 | 9 | DM, CVD, Respiratory | Yes | | Potential benefit | |
| Graham,2016[40] | This systematic review aimed to collate all ACT interventions with chronic disease/long-term conditions, evaluate their quality, and comment on efficacy. | Inception-2015 | 18 | DM, Respiratory, Mental health, Kidney disease, Cerebral Palsy, Brain Injury | Yes | | Potential benefit | |
| Palacio,2016[41] | To evaluate the impact of motivational interviewing (MI) and of the MI delivery format, fidelity assessment, fidelity-based feedback, counselors’ background and MI exposure time on adherence. | 1966-2015 | 17 | Mixed conditions | Yes | | Positive benefit | |
| Eeden,2016[42] | Objective was to systematically review the literature of full economic evaluation studies of self-management interventions in adult chronic patients and to  investigate their methodological quality and cost-effectiveness. | 1990-2014 | 22 | Mixed conditions, DM, Respiratory | Yes | | Positive benefit | |
| Kuo,2016[43] | To assess the clinical evidence supporting the use of secure messaging in EHRs in self-management of diabetes. | NR-2015 | 11 | DM | No | | Potential benefit | |
| Jacelon,2016[44] | The purpose of this scoping review of literature is to explore the types of computer-based systems used for self-management of chronic disease, the goals and success of these systems, the value added by technology integration and the target audience for these systems. | 2006-2016 | 30 | Mixed conditions |  | | Benefit category:  Not applicable | |
| Smith,2017[45] | This review aimed to identify and summarize the existing evidence on the effectiveness of interventions to improve clinical and mental health outcomes and patient-reported outcomes including health-related quality of life for people with multimorbidity in | 1990-2015 | 18 | Multiple chronic conditions | Yes | | Unclear benefit | |
| Laukner,2016[46] | A scoping review was undertaken to discover community-based peer support initiatives for adults in rural settings living with chronic conditions | 2000-2014 | 13 | Mixed  chronic conditions, DM, CVD, Mental health | No | | Positive benefit | |
| Kim,2016[47] | Performed a systematic review to synthesize evidence concerning the types of CBHW interventions, the qualification and characteristics of CBHWs, and patient outcomes and cost-effectiveness of such interventions in vulnerable populations with chronic, non- | Not mentioned-2014 | 67 | CVD, Respiratory, Cancer or cancer screening, Mental health, | Yes | | Positive benefit | |
| Ha Dinh,2016[48] | This systematic review examined the evidence on using the teach-back method in health education programs for improving adherence and self-management of people with chronic disease | Inception-2013 | 10 | DM, CVD, Respiratory | Yes | | Potential benefit | |
| Thakkar,2016[49] | To conduct a meta-analysis of randomized clinical trials to assess the effect of mobile telephone text messaging on medication adherence in chronic disease. | Inception-2015 | 16 | CVD, Respiratory, HIV, allergies, neuro | Yes | | Positive benefit | |
| Deek,2016[50] | To identify elements of effective family-centred self-care interventions that are likely to improve outcomes of adults living with chronic conditions | 2000-2014 | 10 | DM, CVD, Respiratory, Cancer or cancer screening, Musculoskeletal | Yes | | Potential benefit | |
| Kim,2015[51] | This systematic review is aimed at identifying the general characteristics of web-based self-management support inter- ventions for cancer survivors and to perform the correspond- ing meta-analyses to assess the effects of these interventions | 2000-2014 | 37 | Cancer or cancer screening | Yes | | Positive benefit | |
| Peytremann-Bridevaux,2015[52] | To evaluate the effectiveness of chronic disease management programmes for adults with asthma. | Inception-2014 | 20 | Respiratory, Asthma | Yes | | Positive benefit | |
| Jones,2015[53] | The purpose of this review is to evaluate the efficacy of self-management programs in increasing physical activity levels in adults living in the community following acquired brain injury | Inception-2014 | 5 | Neuro, Post-TBI, stroke patients | Yes | | Unclear benefit | |
| Price,2015[54] | Describe the evidence for benefit from patient health record-enabled management, by health condition. | 2008-2014 | 23 | Mixed conditions, DM | No | | Positive benefit | |
| Wildevuur,2015[55] | Aim is to know which ICT interventions have been used to support patients and health care professionals in PCC management of the big 5 chronic diseases and what is the impact of these interventions, such as on health-related quality of life and cost efficiency? | 1989-2013 | 350 | DM, CVD, Respiratory, Cancer or cancer screening, Neuro | No | | Potential benefit | |
| Hamine,2015[56] | To evaluate the effectiveness of mHealth in supporting the adherence of patients to chronic diseases management (“mAdherence”), and the usability, feasibility, and acceptability of mAdherence tools | 1980-2014 | 107 | DM, CVD, Respiratory | No | | Potential benefit | |
| Kruse,2015[57] | Describe the characteristics of portals associated with positive perception by patients and providers. | 2004-2014 | 27 | Mixed  chronic conditions | No | | Unclear benefit | |
| Hammer,2015[58] | The purpose of this integrative review is to evaluate intervention studies led by nurse principal investigators for self-care management in patients with cancer | 2000-2012 | 46 | Cancer or cancer screening | No | | Potential benefit | |
| Kruse,2015[59] | Describe effect of patient portals on quality of care and chronic disease outcomes | 2011-2014 | 27 | Mixed conditions | No | | Unclear benefit | |
| Chi,2015[60] | The present study aimed to systematically review evidence on the effect of telehealth applications on family caregivers | Not mentioned-2014 | 65 | DM, Mental health, Kidney disease, Parkinson disease | Yes | | Positive benefit | |
| Zhao,2015[61] | To examine the effectiveness of telemedicine in relieving asthma symptoms. | Inception-2013 | 11 | Respiratory | Yes | | No benefit | |
| Hill,2015[62] | To systematically review health coaching interventions regarding effectiveness of health coaching for specific outcomes, optimal intervention approaches, and identification of specific techniques associated with effectiveness. | 2000-2012 | 16 1 | Mixed  chronic conditions | Yes | | Positive benefit | |
| Almutairi,2019[63] | To assess the effectiveness of patient activation intervention on T2DM glycemic control and Self-management behaviors SMBs. | 2004-2018 | 10 | DM | No | | Positive benefit | |
| Noonan,2019[64] | To quantify the impact of involving caregivers in self‐management interventions on health‐related quality of life of patients with heart failure or chronic obstructive pulmonary disease. | 1990-2018 | 26 | CVD, Respiratory | Yes | | Benefit category:  Not applicable | |
| Ammenwerth,2019[65] | Assess the effect of patient portals on patient empowerment and health-related outcomes. | 2000-2017 | 10 | Mixed conditions | No | | Unclear benefit | |
| Dendere,2019[66] | Assess effect of inpatient patient portals on patient engagement, health care delivery | 2005-2017 | 58 | Mixed conditions | No | | Unclear benefit | |
| Warrington,2019[67] | This review aimed to (1) describe the features and functions of existing electronic symptom reporting systems (eg, symptom monitoring, tailored self-management advice), and (2) explore which features may be associated with patient engagement and patient-centered outcomes. | 2000-2016 | 29 | Cancer or cancer screening | Yes | | Benefit category:  Not applicable | |
| Boulley,2018[68] | This study aims to highlight the components of Digital health interventions (DI), investigate patient engagement with DI, and explore the effects of DI on psychosocial variables. | Inception-2017 | 29 | Cancer or cancer screening | Yes | | Potential benefit | |
| Kelly,2018[69] | Aim was to review the literature evaluating the design, use, and impact of inpatient portals, which are patient portals designed to give hospitalized patients and caregivers inpatient EHR clinical information for the purpose of engaging them in hospital care. | 2006-2017 | 9 | Mixed conditions | No | | Unclear benefit | |
| Aquino,2017[70] | To identify and evaluate the effectiveness of individual empowerment strategies inpatients with diabetes mellitus (DM). | NR-2016 | 11 | DM | Yes | | No benefit | |
| Donald,2018[71] | To systematically identify and describe self management interventions for adult patients with chronic kidney disease (CKD). | Not mentioned-2016 | 50 | Kidney disease | No | | Unclear benefit | |
| Lee,2018[72] | To examine the effectiveness of mHealth interventions on process measures as well as health outcomes in randomized controlled trials (RCTs) to  improve chronic disease management. | 2005-2016 | 12 | Mixed conditions | Yes | | Positive benefit | |
| Risling,2017[73] | To explore the concept of patient empowerment within the electronic health (eHealth) context | 2000-2016 | 19 | Mixed conditions | No | | Unclear benefit | |
| Pamungkas,2017[74] | This SR aimed to describe the impact of diabetes Self management education that involves family members on patient outcomes related to patient health behaviors such as blood glucose monitoring, medication adherence, lifestyle changes, and physiological markers | 2008-2016 | 23 | DM | No | | Positive benefit | |
| Palacios,2017[75] | To conduct a systematic review to (1) determine the effectiveness of Internet-delivered CHD self-management support for improving CHD, mood, and self-management related outcomes and (2) identify and describe essential components for effectiveness. | 2000-2015 | 7 | CVD, Patients with coronary heart disease (CHD) | Yes | | Potential benefit | |
| Barello,2016[76] | This paper aimed at reviewing findings from the literature about the use of eHealth in engaging patients in their own care process | 2004-2014 | 11 | Mental Health, HIV | No | | Unclear benefit | |
| Pereira,2015[77] | The purpose of this article is to review various delivery methods of Internet diabetes education that have been evaluated, as well as their effectiveness in improving diabetes-related outcomes. (Diabetes self-management education) | 2004-2013 | 14 | DM | No | | Positive benefit | |
| Warner,2015[78] | To identify self-management support strategies in stroke self management interventions and effectively improved outcomes, focusing specifically on function and participation outcomes. | 1986-2012 | 95 | Stroke, neuro | Yes | | Potential benefit | |
| Ammenwerth,2019[65] | To assess the effect of patient portals on patient empowerment and health-related outcomes. | 2000-2017 | 10 | Mixed conditions | No | | Unclear benefit | |
| Banchoff, Janjua S., 2021[79] | To assess benefits and harms of digital interventions for managing COPD and apply Behaviour Change Technique (BCT) taxonomy to  describe and explore intervention content. | Before April 2020 | 14 | Respiratory | Yes | | Unclear benefit | |
| Barbosa, Hannah Cardoso, 2021[80]^[[1]](#footnote-2)^ | To identify empowerment-oriented strategies focused on behavioral change in patients with chronic diseases. | Any period-2020 | 25 | Mix of conditions included | No | | Potential benefit | |
| Hosseinzadeh, Hassan, 2020[81]^[[2]](#footnote-3)^ | This systematic review and meta-analysis aims to address this conflicting evidence by assessing most current randomised controlled trials (RCTs) examining the effect of patient activation embedded programs on diabetes self-management and clinical outcomes compared with usual care among adult individuals (aged .18 years) living with T2DM. | 2004-2019 | 10 | Diabetes | No | | Potential benefit | |
| Smalley, Katelyn R. 2020[82] | The study aims to evaluate the ability of self-management programmes to change the healthcare-seeking behaviours of people with Chronic Obstructive Pulmonary Disease (COPD), and any associations between programme design and outcomes. | 1998-2020 | 26 | Respiratory | Yes | | Unclear benefit | |
| Lin, Mei-Yu, 2020[83] | We conducted a meta-analysis of randomized controlled trials to quantify the outcomes of physiological, psychological, behavioral, and health-related quality of life (HRQOL) for patient activation interventions across these four chronic disease categories. | 2005-2017 | 26 | Diabetes, Cancer or cancer screening, Respiratory, CVD and hypertension | No | | Positive benefit | |
| Yadav, Uday Narayan, 2020[84]^[[3]](#footnote-4)^ | To examine the health literacy and patient activation elements of self-management interventions for Chronic Obstructive Pulmonary Diseases (COPD) patients. | 2008-2019 | 27 | Respiratory | No | | Potential benefit | |
| Pouls, Bart, 2021[85]^[[4]](#footnote-5)^ | (1) to evaluate effectiveness of recently developed and tested interactive eHealth (including mHealth) interventions on medication adherence in adult patients using long-term medication and (2) to describe strategies among effective interventions. | 2014-2019 | 22 | Mix of conditions included | Yes | | Positive benefit | |
| **Shared Decision Making** | | | | | | | |  |
| Voruganti,2017[86] | To conduct a systematic search of the published literature and the Internet for Web-based tools for text-based communication between patients and providers, and map tool characteristics, their intended use, contexts in which they were used, and by whom. | Not mentioned-2016 | 54 | DM, CVD, Respiratory, Mental health, chronic pain, cerebral palsy, eczema | No | | Benefit category:  Not applicable | |
| Vermunt,2017[87] | To identify and evaluate studies on the effects of interventions that support collaborative goal setting or health priority setting compared to usual care for elderly people with a chronic health condition or multimorbidity. | 1990-2015 | 8 | Multiple chronic conditions | Yes | | Positive benefit | |
| Kashaf,2017[88] | This review systematically examines, summarizes and, where possible, quantitatively synthesizes the evidence association between treatment shared decision-making (SDM) and outcomes in diabetes. | 1990-2016 | 16 | DM | Yes | | Positive benefit | |
| Wagner,2019[89] | This review analyzes intervention and evaluation studies on patient education and continuing medical education which aim to enhance shared decision-making. | 2006-2016 | 16 | Mixed chronic conditions | Yes | | Unclear benefit | |
| Goodridge,2019[90] | To map the existing literature and describe interventions aimed at building the capacity of patients to participate in care during hospitalization by: (1) describing and categorizing the aspects of care targeted by these interventions and (2) identifying the behaviour change techniques (BCTs) used in these interventions. | Inception-2017 | 87 | Hospitalized patients | No | | Potential benefit | |
| Baik,2018[91] | The aim of this systematic literature review is to detail and compare interventions supporting SDM over the last 10 years (January 2008 to December 2017) and to analyze patient/caregiver outcomes at the end of life. | 2008-2017 | 12 | End of life | Yes | | Unclear benefit | |
| Berlin,2018[92] | This systematic review and meta-analysis assess the feasibility and efficacy of preoperative decision aids (DAs) to improve the patient decision-making process for breast reconstruction. | Inception-2018 | 17 | Surgical patients | Yes | | Positive benefit | |
| Johnson,2018[93] | To determine the effectiveness of interventions to support shared decision making in hypertension. | Inception-2017 | 11 | Hypertension | Yes | | Unclear benefit | |
| Martínez-González,2018[94] | We sought to evaluate the evidence on the effectiveness of SDM as compared to current clinical practice for patient- and SDM-related outcomes. We focused on the population of men facing preference-sensitive decisions for Prostate Cancer screening. | Inception-2015 | 4 | Cancer or cancer screening | Yes | | No benefit | |
| Légaré,2018[95] | To determine the effectiveness of interventions for increasing the use of SDM by healthcare professionals. We considered interventions targeting patients, interventions targeting healthcare professionals, and interventions targeting both. | 1974-2017 | 87 | Mixed chronic conditions | Yes | | Unclear benefit | |
| Baptista,2018[96] | This meta-analysis aimed to investigate the impact of using Web-based decision aids to support men’s prostate cancer screening decisions in comparison with usual care and other formats of decision aids. | Inception-2016 | 7 | Cancer or cancer screening | Yes | | Potential benefit | |
| Spronk,2018[97] | The aim of this review was to assess the availability and effectiveness of tools supporting SDM in metastatic breast cancer care. | 2006-2017 | 7 | Cancer or cancer screening | Yes | | Potential benefit | |
| Winston,2017[98] | This study reviews the published literature on the use of video-based decision aids (DA) for patients. | NR-2016 | 488 | Mixed chronic conditions,  non-chronic conditions | No | | Potential benefit | |
| Samalin,2018[99] | This study aims to provide a review of the randomized controlled studies evaluating the effects of share decision-making (SDM) intervention in comparison to care as usual in patients with mood disorders. | 2000-2017 | 14 | Mental health, Depression and Bipolar disorder | No | | Positive benefit | |
| Martínez-González,2018[100] | We systematically reviewed the extent of SDM implementation in interventions to facilitate SDM for prostate cancer screening and treatment. | Inception-2015 | 36 | Cancer or cancer screening | No | | Unclear benefit | |
| Martínez-Alonso,2017[101] | The aim of this systematic review and meta-analysis of RCTs and observational studies is to assess the effect of decision aids (DAs) in women aged 50 and below facing the decision to be screened for breast cancer. | Not mentioned-2016 | 4 | Cancer or cancer screening | Yes | | Potential benefit | |
| Kew,2017[102] | To assess benefits and potential harms of shared decision-making for adults and children with asthma. | Not mentioned but first RCT was 1998-2016 | 4 | Respiratory | Yes | | Potential benefit    . | |
| Stacey D,2017[103] | SR to assess the effects of decision aids in people facing treatment or screening decisions. Participants include adults aged 18 years or older who were making decisions about screening or treatment options for themselves, a child, or an incapacitated significant other. | 2012-2015 | 105 | Mixed chronic conditions | Yes | | Positive benefit | |
| Morrell,2016[104] | To describe the range of decision aids (DAs) available to enable informed choice for older patients at the end of life (EOL) and assess their effectiveness or acceptability. | 1995-2015 | 17 | End of life | Yes | | Positive benefit | |
| Porter,2016[105] | The published literature was systematically reviewed to determine the effect of using mobile electronic devices to record food or nutrient intake on diabetes control and nutrition outcomes | Inception-2016 | 9 | DM | Yes | | Unclear benefit | |
| Stovell,2016[106] | To examine the effects of shared decision-making on indices of treatment-related empowerment of people with psychosis | 1806-2015 | 11 | Mental health, | Yes | | Potential benefit | |
| Nathan,2016[107] | We conducted a systematic re- view to characterize the application and effectiveness of DAs in racial, ethnic, sexual, and gender minorities | 2004-2013 | 19 | Mixed chronic conditions, Cancer or cancer screening, kidney disease | Yes | | Positive benefit | |
| Clayman,2015[108] | Assess the extent to which patient participation in decision making within medical encounters is associated with measured patient outcomes. | Inception-2015 | 116 | Mixed  chronic conditions, Non-chronic conditions | Yes | | Positive benefit | |
| van Weert,2016[109] | We sought to systematically review randomized controlled trials (RCTs) and clinical controlled trials (CCTs) evaluating the efficacy of decision aids as compared to usual care or alternative intervention(s) for older adults facing treatment and screening | Inception-2014 | 22 | Mixed chronic conditions | Yes | | Potential benefit | |
| Friedrichs,2016[110] | The aim is therefore to give a systematic overview of the literature of patient preferences and SDM in the treatment of patients with SUD | 1980-2013 | 25 | Mental health | Yes | | Potential benefit | |
| Kashaf,2015[111] | Aims was to review the literature, exploring the association between SDM with regard to treatment and QOL outcomes in cancer, and to identify the variables that moderate this association. | 1992-2014 | 17 | Cancer or cancer screening | Yes | | Potential benefit | |
| McIntyre,2015[112] | This review aims to establish whether computer-based learning systems (CBLSs) should replace standard education for cancer populations. | 2009-2013 | 8 | Cancer or cancer screening | No | | Potential benefit | |
| ElKefi, Safa, 2021[113] | To ensure the well-being of their patients, health care providers (HCPs) are putting more effort into the quality of the communication they provide in oncology clinics. With the emergence of Health Information Technology (HIT), the dynamics between doctors and patients in oncology settings have changed. The purpose of this literature review is to explore and demonstrate how various health information technologies impact doctorpatient communication in oncology settings. | 2009-2020 | 31 | Cancer or cancer screening | No | | Positive benefit | |
| Ammenwerth, Elske, 2021[114] | To assess the effects of providing adult patients with access to electronic health records (EHRs) alone or with additional functionalities on  a range of patient, patient-provider, and health resource consumption outcomes, including patient knowledge and understanding, patient  empowerment, patient adherence, patient satisfaction with care, adverse events, health-related quality of life, health-related outcomes,  psychosocial health outcomes, health resource consumption, and patient-provider communication. | 2017-2020 | 10 | Mix of conditions included | Yes | | Unclear benefit | |
| Fisher, Alana, 2021[115] | The current systematic review aimed to provide a narrative synthesis of the published qualitative and quantitative literature on SDM among adults, either in treatment or in the community, with problematic AOD use and co-occurring mental health conditions | 2000 -2019 | 10 | Multiple conditions, Mental health, Other: Alcohol/other drug use | Yes | | Potential benefit | |
| Butterworth, JE, 2019[116] | To assess the effects of interventions for older patients with multi-morbidity aiming to involve them in decision-making about their health care during primary care consultations. | Up until 2018 | 3 | Multiple conditions | Yes | | Unclear benefit | |
| Mathijssen, Elke, 2019[117] | 1) To examine the effectiveness of interventions to support shared decision making (SDM) for medication therapy in long term conditions on patient outcomes; 2) to identify characteristics of SDM interventions that are associated with positive patient outcomes. | Up to 2019 | 23 | Mix of conditions included | Yes | | Potential benefit | |
| Negarandeh, Reza, 2021[118] | To investigate the effects of question prompt list on shared decision making among cancer patients | 2021 | 2 | Cancer or cancer screening | Yes | | Unclear benefit | |
| Hell, Morten Ellegaard, 2020[119] | to measure outcome results on the following four measures: (1) drinking outcome, (2) quality of life, (3) enrollment, and (4) adherence. | Until Jan. 2019 | 6 | Other: Alcohol addiction | Yes | | Unclear benefit | |
| Keinki, Christian, 2021[120] | To provide a systematic review on the effects of question prompt lists (QPL) in oncological settings. | Any period-July 2019 | 10 | Cancer or cancer screening | Yes | | Unclear benefit | |
| Geddis-Regan, Andrew, 2020[121]^[[5]](#footnote-6)^ | To know which interventions are effective in improving shared decision making or surrogate decision making in relation to the health care of  PLwD. | Not mentioned, search occurred in Dec. 2019 and updated in July 2020 | 8 | Other: Dementia | Yes | | No benefit | |
| DeRosa, Antonio P., 2021[122]^[[6]](#footnote-7)^ | To describe the types of decision-making support interventions offered to racial and ethnic minority adults diagnosed with breast or prostate cancer and to draw any associations between these interventions and patient-reported quality of life (QoL) outcomes | Done on July 2020 | 10 | Cancer or cancer screening | No | | Positive benefit | |
| **Transitional Care** | | | | | | | |  |
| Berre,2017[123] | To determine the effectiveness of interventions targeting transitions from hospital to the primary care setting for chronically ill older patients. | 1995-2016 | 92 | Mixed  chronic conditions | Yes | | Positive benefit | |
| Sendall,2016[124] | This structured review will identify the components of the chronic care model (CCM) required to support healthcare that transitions seamlessly between hospital and ambulatory settings for people over 65 years of age who have two or more chronic diseases | Inception-2015 | 4 | Multiple chronic conditions | Yes | | Positive benefit | |
| **Advanced care planning** | | | | | | | |  |
| Coulter,2015[125] | To assess the effects of personalized care planning for adults with long-term health conditions compared to usual care (i.e. forms of care in which active involvement of patients in treatment and management decisions is not explicitly attempted or achieved | Inception-2013 | 19 | DM, CVD, Respiratory, Mental health, Kidney disease | Yes | | Positive benefit | |
| Nishiwaka, Yuri, 2020[126] | To assess the effects of advance care planning (ACP) in people with heart failure compared to usual care strategies that do not have any components promoting ACP | Not mentioned; search occurred in 2019 | 9 | CVD and hypertension | Yes | | Unclear benefit | |
| **Other** | | | | | | | |  |
| Gershkowitz, Bradley and Hilbert, Connor, 2021[127] | Focused on the clinical impact of digital tools  for providing health coaching, education, and facilitating behavior in patients with  prediabetes or type 2 diabetes. Our approach was designed to provide insights for clinicians and health care systems that are considering adopting such digital tools | 2014-2019 | 21 | Diabetes | Yes | | Positive benefit | |
| Schaepe,2015[128] | To review the current literature on educational interventions used in peritoneal dialysis (PD). | 2006-2013 | 18 | Kidney disease | No | | Unclear benefit | |

**References**

[1] Yin AL, Hachuel D, Pollak JP, Scherl EJ, Estrin D. Digital Health Apps in the Clinical Care of Inflammatory Bowel Disease: Scoping Review. Journal of medical Internet research 2019;21(8):e14630.

[2] Debon R, Coleone JD, Bellei EA, De Marchi ACB. Mobile health applications for chronic diseases: A systematic review of features for lifestyle improvement. Diabetes & metabolic syndrome 2019;13(4):2507-12.

[3] Luedke MW, Blalock DV, Lewinski AA, Shapiro A, Drake C, Lewis JD, et al. VA Evidence-based Synthesis Program Reports. Self-management of Epilepsy: A Systematic Review. Washington (DC): Department of Veterans Affairs (US); 2019.

[4] Warner G, Packer TL, Kervin E, Sibbald K, Audulv A. A systematic review examining whether community-based self-management programs for older adults with chronic conditions actively engage participants and teach them patient-oriented self-management strategies. Patient education and counseling 2019.

[5] Han HR, McKenna S, Nkimbeng M, Wilson P, Rives S, Ajomagberin O, et al. A Systematic Review of Community Health Center Based Interventions for People with Diabetes. Journal of community health 2019.

[6] Levengood TW, Peng Y, Xiong KZ, Song Z, Elder R, Ali MK, et al. Team-Based Care to Improve Diabetes Management: A Community Guide Meta-analysis. American journal of preventive medicine 2019;57(1):e17-e26.

[7] Yin K, Laranjo L, Tong HL, Lau AY, Kocaballi AB, Martin P, et al. Context-Aware Systems for Chronic Disease Patients: Scoping Review. Journal of medical Internet research 2019;21(6):e10896.

[8] Lederle M, Bitzer EM. A close look at lay-led self-management programs for chronic diseases and health care utilisation: A systematic review and meta-analysis. German medical science : GMS e-journal 2019;17:Doc03.

[9] Nazarov S, Manuwald U, Leonardi M, Silvaggi F, Foucaud J, Lamore K, et al. Chronic Diseases and Employment: Which Interventions Support the Maintenance of Work and Return to Work among Workers with Chronic Illnesses? A Systematic Review. International journal of environmental research and public health 2019;16(10).

[10] Sangrar R, Docherty-Skippen SM, Beattie K. Blended face-to-face and online/computer-based education approaches in chronic disease self-management: A critical interpretive synthesis. Patient education and counseling 2019;102(10):1822-32.

[11] Dounavi K, Tsoumani O. Mobile Health Applications in Weight Management: A Systematic Literature Review. American journal of preventive medicine 2019;56(6):894-903.

[12] Skrabal Ross X, Gunn KM, Patterson P, Olver I. Mobile-Based Oral Chemotherapy Adherence-Enhancing Interventions: Scoping Review. JMIR mHealth and uHealth 2018;6(12):e11724.

[13] Lancaster K, Abuzour A, Khaira M, Mathers A, Chan A, Bui V, et al. The Use and Effects of Electronic Health Tools for Patient Self-Monitoring and Reporting of Outcomes Following Medication Use: Systematic Review. Journal of medical Internet research 2018;20(12):e294.

[14] Lycett HJ, Raebel EM, Wildman EK, Guitart J, Kenny T, Sherlock JP, et al. Theory-Based Digital Interventions to Improve Asthma Self-Management Outcomes: Systematic Review. Journal of medical Internet research 2018;20(12):e293.

[15] Bashi N, Fatehi F, Fallah M, Walters D, Karunanithi M. Self-Management Education Through mHealth: Review of Strategies and Structures. JMIR mHealth and uHealth 2018;6(10):e10771.

[16] Alessa T, Abdi S, Hawley MS, de Witte L. Mobile Apps to Support the Self-Management of Hypertension: Systematic Review of Effectiveness, Usability, and User Satisfaction. JMIR mHealth and uHealth 2018;6(7):e10723.

[17] Ko D, Bratzke LC, Roberts T. Self-management assessment in multiple chronic conditions: A narrative review of literature. International journal of nursing studies 2018;83:83-90.

[18] Changizi M, Kaveh MH. Effectiveness of the mHealth technology in improvement of healthy behaviors in an elderly population-a systematic review. mHealth 2017;3:51.

[19] Kelly C, Grundy S, Lynes D, Evans DJ, Gudur S, Milan SJ, et al. Self-management for bronchiectasis. The Cochrane database of systematic reviews 2018;2:Cd012528.

[20] Niznik JD, He H, Kane-Gill SL. Impact of clinical pharmacist services delivered via telemedicine in the outpatient or ambulatory care setting: A systematic review. Research in social & administrative pharmacy : RSAP 2018;14(8):707-17.

[21] Jeddi FR, Nabovati E, Amirazodi S. Features and Effects of Information Technology-Based Interventions to Improve Self-Management in Chronic Kidney Disease Patients: a Systematic Review of the Literature. Journal of medical systems 2017;41(11):170.

[22] Conway CM, Kelechi TJ. Digital Health for Medication Adherence in Adult Diabetes or Hypertension: An Integrative Review. JMIR diabetes 2017;2(2):e20.

[23] Kim BY, Lee J. Smart Devices for Older Adults Managing Chronic Disease: A Scoping Review. JMIR mHealth and uHealth 2017;5(5):e69.

[24] Cho H, Iribarren S, Schnall R. Technology-Mediated Interventions and Quality of Life for Persons Living with HIV/AIDS. A Systematic Review. Applied clinical informatics 2017;8(2):348-68.

[25] Clarkesmith DE, Pattison HM, Khaing PH, Lane DA. Educational and behavioural interventions for anticoagulant therapy in patients with atrial fibrillation. The Cochrane database of systematic reviews 2017;4:Cd008600.

[26] Massimi A, De Vito C, Brufola I, Corsaro A, Marzuillo C, Migliara G, et al. Are community-based nurse-led self-management support interventions effective in chronic patients? Results of a systematic review and meta-analysis. PloS one 2017;12(3):e0173617.

[27] Zomahoun HTV, Guenette L, Gregoire JP, Lauzier S, Lawani AM, Ferdynus C, et al. Effectiveness of motivational interviewing interventions on medication adherence in adults with chronic diseases: a systematic review and meta-analysis. International journal of epidemiology 2017;46(2):589-602.

[28] Whiteman KL, Naslund JA, DiNapoli EA, Bruce ML, Bartels SJ. Systematic Review of Integrated General Medical and Psychiatric Self-Management Interventions for Adults With Serious Mental Illness. Psychiatric services (Washington, DC) 2016;67(11):1213-25.

[29] Zhao FF, Suhonen R, Koskinen S, Leino-Kilpi H. Theory-based self-management educational interventions on patients with type 2 diabetes: a systematic review and meta-analysis of randomized controlled trials. Journal of advanced nursing 2017;73(4):812-33.

[30] Teljeur C, Moran PS, Walshe S, Smith SM, Cianci F, Murphy L, et al. Economic evaluation of chronic disease self-management for people with diabetes: a systematic review. Diabetic medicine : a journal of the British Diabetic Association 2017;34(8):1040-9.

[31] Van Hecke A, Heinen M, Fernandez-Ortega P, Graue M, Hendriks JM, Hoy B, et al. Systematic literature review on effectiveness of self-management support interventions in patients with chronic conditions and low socio-economic status. Journal of advanced nursing 2017;73(4):775-93.

[32] van Hooft SM, Been-Dahmen JMJ, Ista E, van Staa A, Boeije HR. A realist review: what do nurse-led self-management interventions achieve for outpatients with a chronic condition? Journal of advanced nursing 2017;73(6):1255-71.

[33] Stenberg U, Haaland-Overby M, Fredriksen K, Westermann KF, Kvisvik T. A scoping review of the literature on benefits and challenges of participating in patient education programs aimed at promoting self-management for people living with chronic illness. Patient education and counseling 2016;99(11):1759-71.

[34] Plow M, Mangal S, Geither K, Golding M. A Scoping Review of Tailored Self-management Interventions among Adults with Mobility Impairing Neurological and Musculoskeletal Conditions. Frontiers in public health 2016;4:165.

[35] Kew KM, Cates CJ. Home telemonitoring and remote feedback between clinic visits for asthma. The Cochrane database of systematic reviews 2016(8):Cd011714.

[36] Sakakibara BM, Kim AJ, Eng JJ. A Systematic Review and Meta-Analysis on Self-Management for Improving Risk Factor Control in Stroke Patients. International journal of behavioral medicine 2017;24(1):42-53.

[37] van Het Bolscher-Niehuis MJ, den Ouden ME, de Vocht HM, Francke AL. Effects of self-management support programmes on activities of daily living of older adults: A systematic review. International journal of nursing studies 2016;61:230-47.

[38] Sokol R, Fisher E. Peer Support for the Hardly Reached: A Systematic Review. American journal of public health 2016;106(7):e1-8.

[39] Whitehead L, Seaton P. The Effectiveness of Self-Management Mobile Phone and Tablet Apps in Long-term Condition Management: A Systematic Review. Journal of medical Internet research 2016;18(5):e97.

[40] Graham CD, Gouick J, Krahe C, Gillanders D. A systematic review of the use of Acceptance and Commitment Therapy (ACT) in chronic disease and long-term conditions. Clinical psychology review 2016;46:46-58.

[41] Palacio A, Garay D, Langer B, Taylor J, Wood BA, Tamariz L. Motivational Interviewing Improves Medication Adherence: a Systematic Review and Meta-analysis. Journal of general internal medicine 2016;31(8):929-40.

[42] van Eeden M, van Heugten CM, van Mastrigt GA, Evers SM. ECONOMIC EVALUATION STUDIES OF SELF-MANAGEMENT INTERVENTIONS IN CHRONIC DISEASES: A SYSTEMATIC REVIEW. International journal of technology assessment in health care 2016;32(1-2):16-28.

[43] Kuo A, Dang S. Secure Messaging in Electronic Health Records and Its Impact on Diabetes Clinical Outcomes: A Systematic Review. Telemedicine journal and e-health : the official journal of the American Telemedicine Association 2016;22(9):769-77.

[44] Jacelon CS, Gibbs MA, Ridgway JV. Computer technology for self-management: a scoping review. Journal of clinical nursing 2016;25(9-10):1179-92.

[45] Smith SM, Wallace E, O'Dowd T, Fortin M. Interventions for improving outcomes in patients with multimorbidity in primary care and community settings. The Cochrane database of systematic reviews 2016;3:Cd006560.

[46] Lauckner HM, Hutchinson SL. Peer support for people with chronic conditions in rural areas: a scoping review. Rural and remote health 2016;16(1):3601.

[47] Kim K, Choi JS, Choi E, Nieman CL, Joo JH, Lin FR, et al. Effects of Community-Based Health Worker Interventions to Improve Chronic Disease Management and Care Among Vulnerable Populations: A Systematic Review. American journal of public health 2016;106(4):e3-e28.

[48] Ha Dinh TT, Bonner A, Clark R, Ramsbotham J, Hines S. The effectiveness of the teach-back method on adherence and self-management in health education for people with chronic disease: a systematic review. JBI database of systematic reviews and implementation reports 2016;14(1):210-47.

[49] Thakkar J, Kurup R, Laba TL, Santo K, Thiagalingam A, Rodgers A, et al. Mobile Telephone Text Messaging for Medication Adherence in Chronic Disease: A Meta-analysis. JAMA internal medicine 2016;176(3):340-9.

[50] Deek H, Hamilton S, Brown N, Inglis SC, Digiacomo M, Newton PJ, et al. Family-centred approaches to healthcare interventions in chronic diseases in adults: a quantitative systematic review. Journal of advanced nursing 2016;72(5):968-79.

[51] Kim AR, Park HA. Web-based Self-management Support Interventions for Cancer Survivors: A Systematic Review and Meta-analyses. Studies in health technology and informatics 2015;216:142-7.

[52] Peytremann-Bridevaux I, Arditi C, Gex G, Bridevaux PO, Burnand B. Chronic disease management programmes for adults with asthma. The Cochrane database of systematic reviews 2015(5):Cd007988.

[53] Jones TM, Dean CM, Hush JM, Dear BF, Titov N. A systematic review of the efficacy of self-management programs for increasing physical activity in community-dwelling adults with acquired brain injury (ABI). Systematic reviews 2015;4:51.

[54] Price M, Bellwood P, Kitson N, Davies I, Weber J, Lau F. Conditions potentially sensitive to a personal health record (PHR) intervention, a systematic review. BMC medical informatics and decision making 2015;15:32.

[55] Wildevuur SE, Simonse LW. Information and communication technology-enabled person-centered care for the "big five" chronic conditions: scoping review. Journal of medical Internet research 2015;17(3):e77.

[56] Hamine S, Gerth-Guyette E, Faulx D, Green BB, Ginsburg AS. Impact of mHealth chronic disease management on treatment adherence and patient outcomes: a systematic review. Journal of medical Internet research 2015;17(2):e52.

[57] Kruse CS, Argueta DA, Lopez L, Nair A. Patient and provider attitudes toward the use of patient portals for the management of chronic disease: a systematic review. Journal of medical Internet research 2015;17(2):e40.

[58] Hammer MJ, Ercolano EA, Wright F, Dickson VV, Chyun D, Melkus GD. Self-management for adult patients with cancer: an integrative review. Cancer nursing 2015;38(2):E10-26.

[59] Kruse CS, Bolton K, Freriks G. The effect of patient portals on quality outcomes and its implications to meaningful use: a systematic review. Journal of medical Internet research 2015;17(2):e44.

[60] Chi NC, Demiris G. A systematic review of telehealth tools and interventions to support family caregivers. Journal of telemedicine and telecare 2015;21(1):37-44.

[61] Zhao J, Zhai YK, Zhu WJ, Sun DX. Effectiveness of Telemedicine for Controlling Asthma Symptoms: A Systematic Review and Meta-analysis. Telemedicine journal and e-health : the official journal of the American Telemedicine Association 2015;21(6):484-92.

[62] Hill B, Richardson B, Skouteris H. Do we know how to design effective health coaching interventions: a systematic review of the state of the literature. American journal of health promotion : AJHP 2015;29(5):e158-68.

[63] Almutairi N, Hosseinzadeh H, Gopaldasani V. The effectiveness of patient activation intervention on type 2 diabetes mellitus glycemic control and self-management behaviors: A systematic review of RCTs. Primary care diabetes 2019.

[64] Noonan MC, Wingham J, Dalal HM, Taylor RS. Involving caregivers in self-management interventions for patients with heart failure and chronic obstructive pulmonary disease. A systematic review and meta-analysis. Journal of advanced nursing 2019.

[65] Ammenwerth E, Hoerbst A, Lannig S, Mueller G, Siebert U, Schnell-Inderst P. Effects of Adult Patient Portals on Patient Empowerment and Health-Related Outcomes: A Systematic Review. Studies in health technology and informatics 2019;264:1106-10.

[66] Dendere R, Slade C, Burton-Jones A, Sullivan C, Staib A, Janda M. Patient Portals Facilitating Engagement With Inpatient Electronic Medical Records: A Systematic Review. Journal of medical Internet research 2019;21(4):e12779.

[67] Warrington L, Absolom K, Conner M, Kellar I, Clayton B, Ayres M, et al. Electronic Systems for Patients to Report and Manage Side Effects of Cancer Treatment: Systematic Review. Journal of medical Internet research 2019;21(1):e10875.

[68] Escriva Boulley G, Leroy T, Bernetiere C, Paquienseguy F, Desfriches-Doria O, Preau M. Digital health interventions to help living with cancer: A systematic review of participants' engagement and psychosocial effects. Psycho-oncology 2018;27(12):2677-86.

[69] Kelly MM, Coller RJ, Hoonakker PL. Inpatient Portals for Hospitalized Patients and Caregivers: A Systematic Review. Journal of hospital medicine 2018;13(6):405-12.

[70] Aquino JA, Baldoni NR, Flor CR, Sanches C, Di Lorenzo Oliveira C, Alves GCS, et al. Effectiveness of individual strategies for the empowerment of patients with diabetes mellitus: A systematic review with meta-analysis. Primary care diabetes 2018;12(2):97-110.

[71] Donald M, Kahlon BK, Beanlands H, Straus S, Ronksley P, Herrington G, et al. Self-management interventions for adults with chronic kidney disease: a scoping review. BMJ open 2018;8(3):e019814.

[72] Lee JA, Choi M, Lee SA, Jiang N. Effective behavioral intervention strategies using mobile health applications for chronic disease management: a systematic review. BMC medical informatics and decision making 2018;18(1):12.

[73] Risling T, Martinez J, Young J, Thorp-Froslie N. Evaluating Patient Empowerment in Association With eHealth Technology: Scoping Review. Journal of medical Internet research 2017;19(9):e329.

[74] Pamungkas RA, Chamroonsawasdi K, Vatanasomboon P. A Systematic Review: Family Support Integrated with Diabetes Self-Management among Uncontrolled Type II Diabetes Mellitus Patients. Behavioral sciences (Basel, Switzerland) 2017;7(3).

[75] Palacios J, Lee GA, Duaso M, Clifton A, Norman IJ, Richards D, et al. Internet-Delivered Self-management Support for Improving Coronary Heart Disease and Self-management-Related Outcomes: A Systematic Review. The Journal of cardiovascular nursing 2017;32(4):E9-e23.

[76] Barello S, Triberti S, Graffigna G, Libreri C, Serino S, Hibbard J, et al. eHealth for Patient Engagement: A Systematic Review. Frontiers in psychology 2015;6:2013.

[77] Pereira K, Phillips B, Johnson C, Vorderstrasse A. Internet delivered diabetes self-management education: a review. Diabetes technology & therapeutics 2015;17(1):55-63.

[78] Warner G, Packer T, Villeneuve M, Audulv A, Versnel J. A systematic review of the effectiveness of stroke self-management programs for improving function and participation outcomes: self-management programs for stroke survivors. Disability and rehabilitation 2015;37(23):2141-63.

[79] Janjua S, Banchoff E, Threapleton CJ, Prigmore S, Fletcher J, Disler RT. Digital interventions for the management of chronic obstructive pulmonary disease. The Cochrane database of systematic reviews 2021;4(4):Cd013246.

[80] Centers for Disease Control. About Chronic disease. <https://www.cdc.gov/chronicdisease/about/index.htm>.

[81] Hosseinzadeh H, Verma I, Gopaldasani V. Patient activation and Type 2 diabetes mellitus self-management: a systematic review and meta-analysis. Australian journal of primary health 2020;26(6):431-42.

[82] Smalley KR, Aufegger L, Flott K, Mayer EK, Darzi A. Can self-management programmes change healthcare utilisation in COPD?: A systematic review and framework analysis. Patient education and counseling 2020;104(1):50-63.

[83] Lin MY, Weng WS, Apriliyasari RW, P VANT, Tsai PS. Effects of Patient Activation Intervention on Chronic Diseases: A Meta-Analysis. The journal of nursing research : JNR 2020;28(5):e116.

[84] Yadav UN, Lloyd J, Hosseinzadeh H, Baral KP, Harris MF. Do Chronic Obstructive Pulmonary Diseases (COPD) Self-Management Interventions Consider Health Literacy and Patient Activation? A Systematic Review. Journal of clinical medicine 2020;9(3).

[85] Pouls BPH, Vriezekolk JE, Bekker CL, Linn AJ, Onzenoort HAWv, Vervloet M, et al. Effect of Interactive eHealth Interventions on Improving Medication Adherence in Adults With Long-Term Medication: Systematic Review. Journal of medical Internet research 2021;23(1):N.PAG-N.PAG.

[86] Voruganti T, Grunfeld E, Makuwaza T, Bender JL. Web-Based Tools for Text-Based Patient-Provider Communication in Chronic Conditions: Scoping Review. Journal of medical Internet research 2017;19(10):e366.

[87] Vermunt N, Harmsen M, Westert GP, Olde Rikkert MGM, Faber MJ. Collaborative goal setting with elderly patients with chronic disease or multimorbidity: a systematic review. BMC geriatrics 2017;17(1):167.

[88] Saheb Kashaf M, McGill ET, Berger ZD. Shared decision-making and outcomes in type 2 diabetes: A systematic review and meta-analysis. Patient education and counseling 2017;100(12):2159-71.

[89] Wagner A, Radionova N, Rieger MA, Siegel A. Patient Education and Continuing Medical Education to Promote Shared Decision-Making. A Systematic Literature Review. International journal of environmental research and public health 2019;16(14).

[90] Goodridge D, McDonald M, New L, Scharf M, Harrison E, Rotter T, et al. Building patient capacity to participate in care during hospitalisation: a scoping review. BMJ open 2019;9(7):e026551.

[91] Baik D, Cho H, Masterson Creber RM. Examining Interventions Designed to Support Shared Decision Making and Subsequent Patient Outcomes in Palliative Care: A Systematic Review of the Literature. The American journal of hospice & palliative care 2019;36(1):76-88.

[92] Berlin NL, Tandon VJ, Hawley ST, Hamill JB, MacEachern MP, Lee CN, et al. Feasibility and Efficacy of Decision Aids to Improve Decision Making for Postmastectomy Breast Reconstruction: A Systematic Review and Meta-analysis. Medical decision making : an international journal of the Society for Medical Decision Making 2019;39(1):5-20.

[93] Johnson RA, Huntley A, Hughes RA, Cramer H, Turner KM, Perkins B, et al. Interventions to support shared decision making for hypertension: A systematic review of controlled studies. Health expectations : an international journal of public participation in health care and health policy 2018;21(6):1191-207.

[94] Martinez-Gonzalez NA, Neuner-Jehle S, Plate A, Rosemann T, Senn O. The effects of shared decision-making compared to usual care for prostate cancer screening decisions: a systematic review and meta-analysis. BMC cancer 2018;18(1):1015.

[95] Legare F, Adekpedjou R, Stacey D, Turcotte S, Kryworuchko J, Graham ID, et al. Interventions for increasing the use of shared decision making by healthcare professionals. The Cochrane database of systematic reviews 2018;7:Cd006732.

[96] Baptista S, Teles Sampaio E, Heleno B, Azevedo LF, Martins C. Web-Based Versus Usual Care and Other Formats of Decision Aids to Support Prostate Cancer Screening Decisions: Systematic Review and Meta-Analysis. Journal of medical Internet research 2018;20(6):e228.

[97] Spronk I, Burgers JS, Schellevis FG, van Vliet LM, Korevaar JC. The availability and effectiveness of tools supporting shared decision making in metastatic breast cancer care: a review. BMC palliative care 2018;17(1):74.

[98] Winston K, Grendarova P, Rabi D. Video-based patient decision aids: A scoping review. Patient education and counseling 2018;101(4):558-78.

[99] Samalin L, Genty JB, Boyer L, Lopez-Castroman J, Abbar M, Llorca PM. Shared Decision-Making: a Systematic Review Focusing on Mood Disorders. Current psychiatry reports 2018;20(4):23.

[100] Martinez-Gonzalez NA, Plate A, Senn O, Markun S, Rosemann T, Neuner-Jehle S. Shared decision-making for prostate cancer screening and treatment: a systematic review of randomised controlled trials. Swiss medical weekly 2018;148:w14584.

[101] Martinez-Alonso M, Carles-Lavila M, Perez-Lacasta MJ, Pons-Rodriguez A, Garcia M, Rue M. Assessment of the effects of decision aids about breast cancer screening: a systematic review and meta-analysis. BMJ open 2017;7(10):e016894.

[102] Kew KM, Malik P, Aniruddhan K, Normansell R. Shared decision-making for people with asthma. The Cochrane database of systematic reviews 2017;10:Cd012330.

[103] Stacey D, Legare F, Lewis K, Barry MJ, Bennett CL, Eden KB, et al. Decision aids for people facing health treatment or screening decisions. The Cochrane database of systematic reviews 2017;4:Cd001431.

[104] Cardona-Morrell M, Benfatti-Olivato G, Jansen J, Turner RM, Fajardo-Pulido D, Hillman K. A systematic review of effectiveness of decision aids to assist older patients at the end of life. Patient education and counseling 2017;100(3):425-35.

[105] Porter J, Huggins CE, Truby H, Collins J. The Effect of Using Mobile Technology-Based Methods That Record Food or Nutrient Intake on Diabetes Control and Nutrition Outcomes: A Systematic Review. Nutrients 2016;8(12).

[106] Stovell D, Morrison AP, Panayiotou M, Hutton P. Shared treatment decision-making and empowerment-related outcomes in psychosis: systematic review and meta-analysis. The British journal of psychiatry : the journal of mental science 2016;209(1):23-8.

[107] Nathan AG, Marshall IM, Cooper JM, Huang ES. Use of Decision Aids with Minority Patients: a Systematic Review. Journal of general internal medicine 2016;31(6):663-76.

[108] Clayman ML, Bylund CL, Chewning B, Makoul G. The Impact of Patient Participation in Health Decisions Within Medical Encounters: A Systematic Review. Medical decision making : an international journal of the Society for Medical Decision Making 2016;36(4):427-52.

[109] van Weert JC, van Munster BC, Sanders R, Spijker R, Hooft L, Jansen J. Decision aids to help older people make health decisions: a systematic review and meta-analysis. BMC medical informatics and decision making 2016;16:45.

[110] Friedrichs A, Spies M, Harter M, Buchholz A. Patient Preferences and Shared Decision Making in the Treatment of Substance Use Disorders: A Systematic Review of the Literature. PloS one 2016;11(1):e0145817.

[111] Kashaf MS, McGill E. Does Shared Decision Making in Cancer Treatment Improve Quality of Life? A Systematic Literature Review. Medical decision making : an international journal of the Society for Medical Decision Making 2015;35(8):1037-48.

[112] McIntyre R, Craig A. A Literature Review of Patient Education: Is IT Time to Move Forward? Journal of medical imaging and radiation sciences 2015;46(3s):S75-s85.

[113] ElKefi S, Asan O. How technology impacts communication between cancer patients and their health care providers: A systematic literature review. International journal of medical informatics 2021;149:104430.

[114] Ammenwerth E, Neyer S, Hörbst A, Mueller G, Siebert U, Schnell-Inderst P. Adult patient access to electronic health records. The Cochrane database of systematic reviews 2021;2:Cd012707.

[115] Fisher A, Mills K, Teesson M, Marel C. Shared decision-making among people with problematic alcohol/other drug use and co-occurring mental health conditions: A systematic review. Drug and alcohol review 2020;40(2):307-24.

[116] Butterworth JE, Hays R, McDonagh STJ, Richards SH, Bower P,et al. -Interventions for involving older patients with multi‐morbidity in decision‐making during primary care consultations. Cochrane Database of Systematic Reviews 2019, Issue 10. Art. No.: CD013124. DOI: 10.1002/14651858.CD013124.pub2. Accessed 03 October 2021. Patient education and counseling 2020;103(10):2078-94.

[117] Mathijssen EGE, van den Bemt BJF, van den Hoogen FHJ, Popa CD, Vriezekolk JE. Interventions to support shared decision making for medication therapy in long term conditions: A systematic review. Patient education and counseling 2019;103(2):254-65.

[118] Negarandeh R, Yazdani Z, Ramtin S, Janani L. Impact of using question prompt list on shared decision-making in the cancer patient: A systematic review. Nursing Practice Today 2021;8(3):179-93.

[119] Hell ME, Nielsen AS. Does patient involvement in treatment planning improve adherence, enrollment and other treatment outcome in alcohol addiction treatment? A systematic review. Addiction Research & Theory 2020;28(6):537-45.

[120] Sharma AE, Rivadeneira NA, Barr-Walker J, Stern RJ, Johnson AK, Sarkar U. Patient Engagement In Health Care Safety: An Overview Of Mixed-Quality Evidence. Health affairs (Project Hope) 2018;37(11):1813-20.

[121] Geddis-Regan A, Errington L, Abley C, Wassall R, Exley C, Thomson R. Enhancing shared and surrogate decision making for people living with dementia: A systematic review of the effectiveness of interventions. Health expectations : an international journal of public participation in health care and health policy 2020;24(1):19-32.

[122] DeRosa AP, Grell Y, Razon D, Komsany A, Pinheiro LC, Martinez J, et al. Decision-making support among racial and ethnic minorities diagnosed with breast or prostate cancer: A systematic review of the literature. Patient education and counseling 2021.

[123] Le Berre M, Maimon G, Sourial N, Gueriton M, Vedel I. Impact of Transitional Care Services for Chronically Ill Older Patients: A Systematic Evidence Review. Journal of the American Geriatrics Society 2017;65(7):1597-608.

[124] Sendall M, McCosker L, Crossley K, Bonner A. A structured review of chronic care model components supporting transition between healthcare service delivery types for older people with multiple chronic diseases. Health information management : journal of the Health Information Management Association of Australia 2017;46(2):58-68.

[125] Coulter A, Entwistle VA, Eccles A, Ryan S, Shepperd S, Perera R. Personalised care planning for adults with chronic or long-term health conditions. The Cochrane database of systematic reviews 2015(3):Cd010523.

[126] Nishikawa Y, Hiroyama N, Fukahori H, Ota E, Mizuno A, Miyashita M, et al. Advance care planning for adults with heart failure. The Cochrane database of systematic reviews 2020;2(2):Cd013022.

[127] Hillert CJ, Gershkowitz B, Crotty BH. Digital coaching strategies to facilitate behavioral change in type ii diabetes: A systematic review. Journal of general internal medicine 2020;35(SUPPL 1):S645-S6.

[128] Schaepe C, Bergjan M. Educational interventions in peritoneal dialysis: a narrative review of the literature. International journal of nursing studies 2015;52(4):882-98.

1. 1. This reference also contains a health literacy intervention type.

   2. This reference also contains a shared decision making intervention type. [↑](#footnote-ref-2)
2. [↑](#footnote-ref-3)
3. 3. This reference also contains a health literacy intervention type.

   4. This reference also contains a shared decision making intervention type. [↑](#footnote-ref-4)
4. [↑](#footnote-ref-5)
5. 5. This reference also contains an advanced care planning type. [↑](#footnote-ref-6)
6. 6. This reference also contains a health literacy type. [↑](#footnote-ref-7)
